# Metal(loid) bioaccessibility and risk assessment of ashfall deposit from Popocatépetl volcano, Mexico

**DOI:** 10.1007/s10653-024-02135-8

**Published:** 2024-07-30

**Authors:** Benedetto Schiavo, Diana Meza-Figueroa, Ofelia Morton-Bermea, Aracely Angulo-Molina, Belem González-Grijalva, María Aurora Armienta-Hernández, Claudio Inguaggiato, Francisco Berrellez-Reyes, Daisy Valera-Fernández

**Affiliations:** 1 Instituto de Geofísica, Universidad Nacional Autónoma de México, 04510 Mexico City, Mexico; 2 Departamento de Geología, Universidad de Sonora, 83000 Hermosillo, Mexico; 3 Departamento de Ciencias Químico-Biológicas, Universidad de Sonora, 83000 Hermosillo, Mexico; 4 Departamento de Geología, Centro de Investigación Científica y de Educación Superior de Ensenada, Baja California (CICESE), Ensenada, Mexico; 5 Instituto de Geología, Universidad Nacional Autónoma de México, 04510 Mexico City, Mexico

**Keywords:** Heavy metal, Lung bioaccessibility, Gamble solution, Artificial lysosomal fluids, Particle size

## Abstract

Ash emission from volcanic eruptions affects the environment, society, and human health. This study shows the total concentration and lung bioaccessible fraction of eight potential toxic metal(loid)s in five Popocatépetl ashfall samples. Mineralogical phases and particle size distribution of the ashfall were analyzed by X-ray diffraction (XRD) and Scanning Electron Microscope (SEM) techniques, respectively. The bioaccessibility test of Gamble solution (GS) and Artificial Lysosomal Fluid (ALF) were conducted to simulate extracellular (pH 7) and intracellular (pH 4.5) conditions, respectively. The studied metal(loid)s showed the following total concentration (mg kg^−1^): 1.98 (As), 0.17 (Cd), 134.09 (Cr), 8.66 (Cu), 697.33 (Mn), 55.35 (Ni), 8.77 (Pb), and 104.10 (Zn). Geochemical indices suggested that some metal(loid)s are slightly enriched compared to the local soil background concentrations. Several mineralogical phases were identified in the collected ashfall deposits, such as plagioclase, pyroxene, and Fe–Ti oxide, among others. According to the risk assessment results, the non-carcinogenic risk related to ashfall exposure returns an HQ > 1 for children. In contrast, the estimation of carcinogenic risk was found to be within the tolerable limit. Metal(loid)s showed low bioaccessibility (< 30%) in GS and ALF, with the highest values found in ALF solution for As (12.18%) and Cu (7.57%). Despite their metal-bioaccessibility, our findings also showed that dominant ash particle size ranged between fine (< 2.5 μm) and extremely fine (< 1 μm), considered highly inhalable fractions. The results obtained in this work indicate that volcanic ashes are bioinsoluble and biodurable, and exhibit low bioaccessibility when in contact with lung human fluids.

**Supplementary Information:**

The online version contains supplementary material available at 10.1007/s10653-024-02135-8.

## Introduction

Volcanic eruptions are a significant natural source of contaminants, which can directly affect people, livelihoods, and economies locally and worldwide (Tomii et al., 2020). Around 30 and 800 million people live within a 10 and 100 km radius of active volcanoes, respectively (Brown et al., 2015). Furthermore, especially for populations residing near (~ 10 km) an active volcanic area, eruptions involve a considerable social impact that depends on the variability of volcanic activity, tephra dispersion, pyroclastic events, and the population's perception of vulnerability (Barclay et al., 2015; Covey et al., 2021), among other factors. These people in some areas are constantly exposed to gases and aerosols, which represent an environmental and air quality deterioration due to the presence of several potentially toxic elements (Freire et al., 2019). Several authors report the impact of volcanic eruptions on the local atmosphere (Trejos et al., 2021) and even at distances of a thousand of kilometers (Sun et al., 2014). In 2010, the eruption of the Eyjafjallajökull volcano in Iceland caused severe disruptions to the European aviation system and economic activities due to the ash dispersion across the continent (Langmann et al., 2012). Strong volcanic eruptions can induce global climate change by injecting greenhouse gases and aerosols into the troposphere (Ilyinskaya et al., 2021).

Volcanic emissions include various gases, mainly water vapor (H_2_O), carbon dioxide (CO_2_), and sulfur dioxide (SO_2_), which represent more than 90% of the total plume composition (Woitischek et al., 2021). Other gases, present in a minor concentration, include halogens, such as hydrogen chloride (HCl) and hydrogen fluoride (HF), which are highly reactive compounds characterized by a short atmospheric lifetime (Aiuppa et al., 2009; Stremme et al., 2023). Volcanic events are also characterized by release of particles of various sizes, commonly referred to as volcanic ashfall. Generally, the diameter of the ashes is less than 2 mm but recent studies report submicrometric and nanometric size fractions in volcanic ash particles (Ermolin et al., 2018; Schiavo et al., 2023b). Volcanic ash is classified as primary particulate emissions, characterized by different densities, sizes, and compositions. On the other hand, sulfates (originating from sulfur compounds) and metal-bearing aerosols are formed in the atmosphere through gas-to-particle conversion during secondary formation processes (Tomasi & Lupi, 2016). Gaseous species act as carriers for the transport of metals (Mandon et al., 2019), including highly volatile trace substances like Hg (Schiavo et al., 2020a). The formation and emission rate of metal and metalloids (e.g., As, Cd, Zn, Se, Sb, Te, Au, etc.) from volcanic plumes are related to magmatic processes and element-specific volatility. Metal volatility depends on different parameters and pre-eruptive conditions, such as oxidation state, fluids density, trace species concentration, magma fragmentation, pressure, and temperature (Edmonds et al., 2022; Ilyinskaya et al., 2021). The plume of an active volcano continuously releases metals during eruptive phases and quiescent periods, with different flux emissions. Henley and Berger (2013) reported a different enrichment of volcanic trace metals depending on the geodynamic context and tectonic setting (e.g., basaltic, arc, or intraplate volcanoes).

Previously published works described critical health hazards of exposure to volcanic gases and particulate matter (Horwell & Baxter, 2006; Mueller et al., 2020). Exposure to volcanic ash generates eye irritation and inflammation of the upper respiratory tract. Asthma and bronchitis (i.e., acute respiratory diseases) are commonly reported as effects of short-term inhalation of volcanic ash (Lombardo et al., 2013). However, prolonged exposure over years can lead to several types of cancer, silicosis, pulmonary fibrosis, and chronic bronchitis, as well as exacerbate pre-existing lung diseases (Gudmundsson, 2010). The health impact of volcanic depends on the mineralogy, particle size, surface reactivity, and physical–chemical properties. In particular, fine-grained ash (< 2.5 μm in aerodynamic diameter) is considered hazardous for health (Thangavel et al., 2022), because it can easily penetrate into the deep part of the lungs. Besides, ash particles contain bio-toxic trace elements considered carcinogenic and hydroxyl radicals (e.g., **·**OH) that can damage cell components (Horwell, 2007). Toxic metals present in the ash can enter the human body through inhalation and even through ingestion of contaminated water and food. The accumulation and absorption of trace metals in volcanic ash into the human body can cause serious health complications. According to Ferreira et al. (2015), once in contact with biological molecules, toxic metals can induce the generation of reactive oxygen species and DNA (Deoxyribonucleic Acid) deterioration via oxidative stress mechanism (Schiavo et al., 2023a). Several experimental studies report pulmonary (Camarinho et al., 2021), neurological (Navarro-Sempere et al., 2021), and reproductive (Ferreira et al., 2015) damage in mice exposed to volcanic trace metals, particularly Hg (Navarro-Sempere, et al., 2020). Vigneri et al. (2017), in their study of the incidence and speciation of metals in volcanic environments, found a correlation between exposure to trace metals and an increase in thyroid cancer case. Additionally, Amaral et al. (2008) and Varrica et al. (2014) conducted biomonitoring studies on school children chronically exposed to volcanic emissions, in order to describe anomalous concentration of trace metals on the scalp hair.

Recently, the application of simulated lung fluids (SLF) test has been widely used to assess the health risk of trace metal inhalation exposure (Kastury et al., 2018; Meza-Figueroa et al., 2020; Schiavo et al., 2023a), estimating the available fraction of absorption via the respiratory tract through leaching experiments (Tomašek et al., 2021). In the literature (Meza-Figueroa et al., 2020), the most used in vitro pulmonary solutions are: (1) Gamble solution (GS, pH 7) and Artificial Lysosomal Fluids (ALF, pH 4.5). Compared to in vivo studies, in vitro analyses are less expensive, simpler to conduct, and do not require approval from an ethics committee. In volcanic environments, one of the first studies that reports an inflammatory response of respirable magmatic minerals was carried out by Damby et al. (2016), reporting a pro-inflammatory and cytotoxicity condition in the THP-1 (human monocytic cell line) macrophage cells due to exposure to cristobalite. Furthermore, Tomašek et al. (2021) describe a standardized in vitro model for determining volcanic soluble toxic elements. Compared to the extensive research on volcanic gases, mainly sulfur compounds, significant lacks are present about the impact of volcanic trace metals in contact with lung fluids. Despite a wide application in anthropogenic environments (Schiavo et al., 2021), like urban areas or industrial complexes, bioaccessibility is relatively few studied in volcanic ash samples. The main novelty of the article concerns the bioaccessibility of volcanic ash metal(loid)s in the alveolar macrophage region. The behavior of ash particles in contact with pulmonary biological fluids provide information on the durability and solubility of volcanic materials. The bioaccesible fraction of metal(loid)s in volcanic ash is a key factor for risk assessment and particles characterization. Therefore, detailed studies on the concentration of the bioaccessible fraction of trace metals in volcanic ash are necessary.

The objective of this work is to: (1) report the metal(loid) concentrations of ashfall coming from Popocatépetl volcano and provide a comparison with other volcanoes around the world; (2) characterize the particle size distribution using Scanning Electron Microscope (SEM); (3) identify the mineralogical phases by X-ray diffraction (DRX); (4) estimate the health risk exposure to metal(loid) from the volcanic ashfall emissions; and (5) assess the lung bioaccessibility using two in vitro (GS and ALF) lung solutions. This work represents the first report of in vitro pulmonary bioaccessibility of metal(loid)s in extra- and intra-cellular conditions for ashfall deposits from the Popocatépetl volcano.

## Materials and methods

### Study area

Popocatépetl is an andesitic stratovolcano localized in a central part of the geological area called Trans-Mexican Volcanic Belt (TMVB). This magmatic arc was generated by the subduction of two oceanic plates, Cocos and Rivera, beneath the continental North American Plate (Schaaf et al., 2005). With an approximate age of 700,000 years, based on a paleomagnetic study (Conte et al., 2004), the Popocatepetl volcano is one of the most active and largest volcanoes in Latin America, that reaches an altitude of 5500 m above sea level (m.a.s.l) and with an estimated diameter of 25 km. After a quiescence phase, Popocatépetl restarted its activity in December 1994. Since 1994, the new activity has been characterized by persistent passive degassing, pulsating ash emissions, and occasional medium-intensity eruptions, as well as episodes of dome growth and destruction followed by a Vulcanian-type activity. Considering its geological history, Popocatépetl is classified as potentially dangerous mainly due to its proximity to densely populated areas and, therefore, one of the most monitored volcanoes (Witter et al., 2005).

**Fig. 1:**
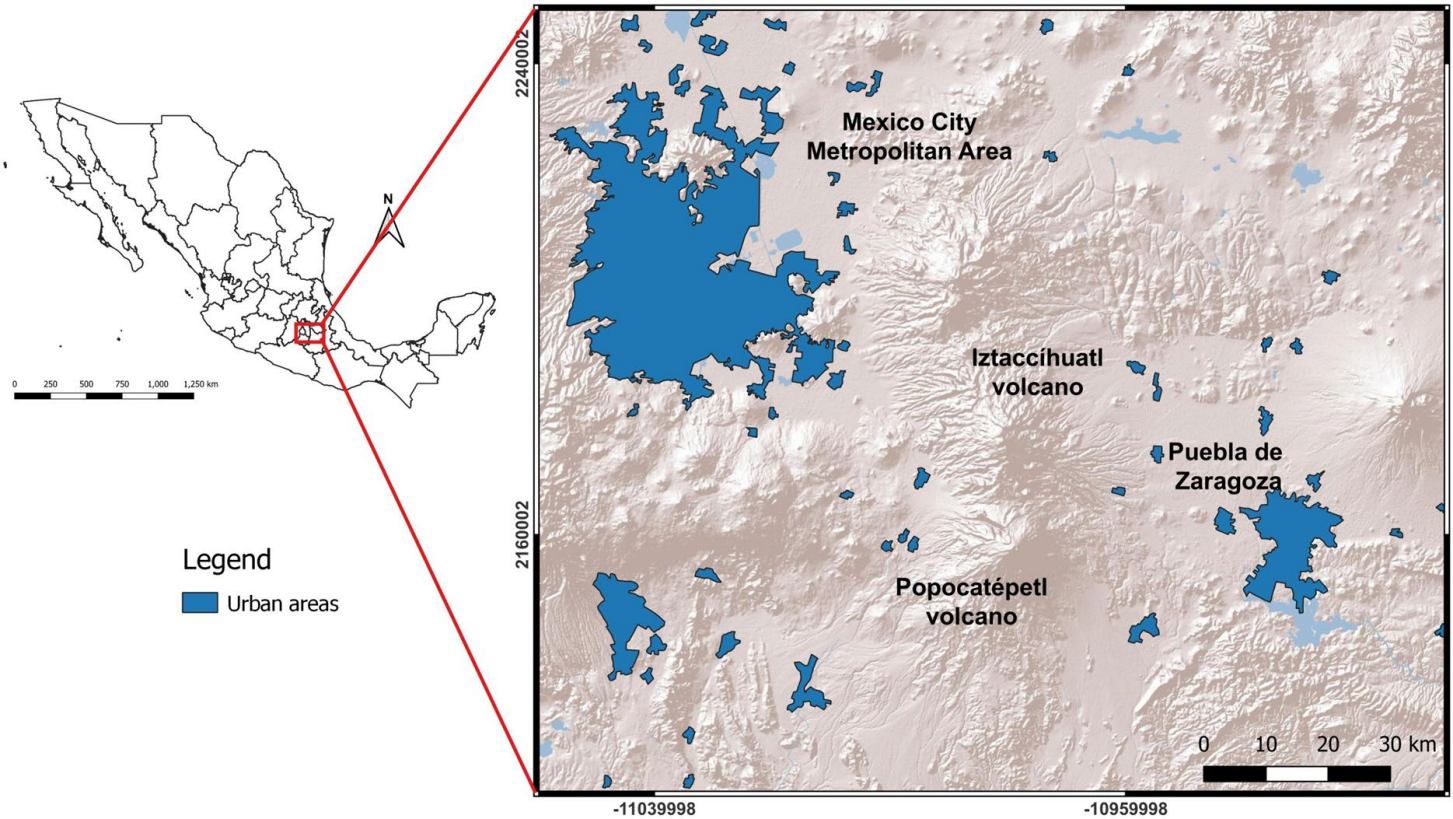
Location map of the Popocatépetl volcano area. The area is situated between two megacities, Mexico City and Puebla de Zaragoza

Popocatépetl volcano is situated in central Mexico (19° 1′ 19″ North, 98° 37′ 40″ West), between the states of Puebla, Morelos, and Estado de México, close to megacities: 70 km south-east of Mexico City (~ 9 million inhabitants) and 45 km west of Puebla de Zaragoza (~ 6.5 million inhabitants) (Fig. 1). Around 150,000 people from different communities live in the volcano area. Moreover, several human activities such as agricultural fields, industrial complexes, and waterways (e.g., Zahuapan-Atoyac River) are located near to the volcano. Currently, volcano surveillance is carried out by the National Center for Disasters Prevention (CENAPRED) in collaboration with academic institutions, using direct and indirect methods. Direct approaches for assessing volcanic risk involve petrological studies, spring waters and fumaroles sampling, and seismic techniques (Werner et al., 1997; Ramos Jiménez, 2019). On the other hand, indirect remote sensing techniques use the physical principles of spectroscopy to remotely estimate the composition and concentration of gases and particles emitted by the volcano (Schiavo et al., 2019, 2020a, b Taquet et al., 2019; Stremme et al., 2023).

### Sample collection and analysis

A total of 5 volcanic ashfall samples were collected on the slopes of the Popocatépetl volcano using high density polyethylene (HDPE) containers at a distance of about 3–5 km from the crater during a low-medium intensity eruption occurred in May 2022. The sampling points are shown in Fig. S1. During the dry period without rain or plume condensation and low ash emissions, approximately 200 g of ashfall samples were collected. After collection, the samples were placed in polyethylene bags and stored at ambient temperature. In the laboratory, the samples were dried in ovens at 25 ºC and then mechanically sieved (200 μm mesh) to separate the coarse particles.

The concentrations of eight metal(loid)s in the ashfall samples (As, Cd, Cr, Cu, Mn, Ni, Pb, and Zn) were evaluated by inductively coupled plasma–mass spectrometry (ICP–MS, model ICAP Qc, Thermo Fisher, USA) at the Institute of Geophysics, National Autonomous University of Mexico (IGF-UNAM). The total digestion procedure involved 0.2 g of ashfall sample and an acid combination of 4 mL HClO_4_ and 10 mL HF. A prepared mixture was placed in Teflon digestion vessels and inserted into a microwave oven (ETHOS ONE) equipped with a rotor system (PRO-24) in order to assist the digestion procedure. Prior to analysis by ICP-MS, the solution was cooled, dried, and diluted with 50 mL of 3% HNO_3_ to determine the metal(loid)s concentration. The calibration curve was obtained with 14 points (0 to 500 μg L^−1^) from a multi-element standard solution (ICP-MS-68A). For quality assurance and control, the accuracy of the procedure was determined using standard reference material 2709A (SRM-2709A), which was analyzed in combination with the ashfall samples. SRM-2709A is a reference material consisting of soil collected from an agricultural area (San Joaquin, California, USA) with a particle size of less than 74 μm. Average recoveries (Table S1) for each metal(loid) were: 85% (As), 102% (Cd), 123% (Cr), 105% (Cu), 114% (Mn), 115% (Ni), 118% (Pb), and 94% (Zn). The detection limits (LD) for the analyzed metal(loid)s, expressed in μg L^−1^, were As = 0.2, Cd = 0.01, Cr = 0.006, Cu = 0.05, Mn = 0.006, Ni = 0.02, Pb = 0.02, and Zn = 0.3. Additionally, methodological blanks and triplicate analysis were performed during the analytical session.

### Mineralogical and particle distribution analyses

Mineralogical analysis was obtained using the X-ray diffraction (XRD) model Bruker D8 Advance, which is especially useful for solid samples. The instrument is equipped with a scan range from 6° to 77° (2theta) and a step size of 0.02° (2 s time per step). Finally, the spectrum interpretation was performed using the Diffrac-plus EVA software.

The particle size distribution was performed using Particle Metric software (PMS) by SEM model Thermo Fisher Scientific Phenom Pro (MA, USA). PMS evaluates several parameters, such as size, equivalent diameter, and circularity of samples. The preparation of ashfall deposits to determine size distribution involves a resuspension chamber and a constant flux of compressed air to resuspend fine particles (i.e., breathable fraction) for simulating external conditions. After a while, the ash particles settle by gravity onto a support positioned inside the SEM. More information related to the experimental procedure of the resuspension chamber can be found in Meza-Figueroa et al. (2020).

### Geochemical indices

Geochemical indices are widely used to determine metal enrichment and potential ecological risk in various environmental matrices like dust, soil, and ash. Three different indices were applied to the volcanic ashfall samples: geoaccumulation index, enrichment factor, and ecological risk index. Geochemical indices require background values to be calculated. In this work, we used background values of Popocatepetl volcanic soils collected in July 2012 reported by Rodriguez-Espinosa et al. (2015). Geochemical background values are listed in Table 1.

**Table 1:** Concentration (mg kg^−1^) of metal(loid)s in ashfall deposit at Popocatépetl volcano

	As	Cd	Cr	Cu	Mn	Ni	Pb	Zn
*Sample*								
M1	1.65	0.25	134.40	10.65	724.68	63.67	8.21	95.94
M2	2.40	0.13	125.70	6.45	650.91	61.25	8.82	102.35
M3	1.89	0.15	151.86	11.91	739.72	53.32	12.08	85.72
M4	2.20	0.12	130.47	6.10	770.45	60.40	6.77	116.96
M5	1.75	0.16	128.04	8.19	599.88	38.13	7.63	119.54
Mean	1.98	0.17	134.09	8.66	697.33	55.35	8.78	104.10
Min	1.65	0.12	125.70	6.10	599.88	38.13	6.77	85.72
Max	2.40	0.25	151.86	11.91	770.45	63.67	12.08	119.54
Median	1.89	0.15	130.47	8.19	725.68	60.40	8.21	102.35
Standard deviation	0.28	0.05	9.34	2.29	62.61	9.28	1.82	12.74
Skewness	0.34	1.16	1.16	0.24	− 0.43	− 1.05	0.99	− 0.09
Local background^a^	1.54	4.67	96	23	950	87.33	60.33	81

^a^Rodriguez-Espinosa et al. (2015)

#### Geoaccumulation index

The geoaccumulation index (I_geo_) was originally proposed by Müller (1969) and used to evaluate the pollution degree comparing the level of metal(loid) obtained from sample to background level. I_geo_ is calculated using the following equation:

1 $$I_{geo} = log_{2} \left( {\frac{{C_{n} }}{{1.5 \cdot B_{n} }}} \right)$$

where *C*_*n*_ is the metal(loid) concentration of ashfall, *B*_*n*_ is the geochemical background value, and 1.5 is the lithospheric background matrix correction. Details on contamination levels of I_geo_ are given in Table S2.

#### Enrichment factor

Enrichment factor (EF) is a useful index with the ability to differentiate between various sources (natural or anthropogenic). The calculation of EF was first proposed by Taylor and McLennan (1985) using the equation as follows:

2 $$EF = \left( {\frac{{C_{n} /C_{ref} }}{{B_{n} /B_{ref} }}} \right)$$

where *C*_*ref*_ is the concentration of the reference element and B_ref_ is the concentration of the reference element in the background. In this work, aluminum (Al) is used as a reference conservative element. The EF contamination level is classified into several categories (Cakmak et al., 2020) which can be found in Table S2.

#### Potential ecological risk assessment

The potential ecological risk index (RI), proposed by Hakanson (1980), is used to assess the risk of metal(loid) to biological and ecological communities exposed to a contaminated source. RI was calculated by the following expression

3 $$RI = \sum E_{f}^{i} = T_{r}^{i} \cdot CF$$

where *RI* is the sum of all metal(loid) risk factors, *E*_*f*_ is the potential ecological risk factor, *CF* is the contamination factor calculated by *C*_*n*_*/B*_*n*_ ratio, and *T*_*r*_ is the toxic response factor representing an environmental sensitivity of specific metal(loid). Hakanson (1980) proposed the response factor of 10, 30, 2, 5, 1, 5, 5, and 1 for As, Cd, Cr, Cu, Mn, Ni, Pb, and Zn, respectively (Maanan et al., 2015). Table S2 shows the different categories that represent the potential ecological risk index.

### Health risk assessment

The non-carcinogenic and carcinogenic health risks associated with metal(loid) exposure in children and adults were investigated using the methodology developed by the United States-Environmental Protection Agency (USEPA) (USEPA, 2001). Average daily dose (ADD) for different pathways, ingestion (ADD_ing_), inhalation (ADD_inh_), and dermal absorption (ADD_der_), was calculated in the USEPA equations:

4 $$ADD_{ing} = \frac{C \times IngR \times EF \times ED}{{BW \times AT_{nc \left( c \right)} }} \times 10^{ - 6}$$

5 $$ADD_{inh} = \frac{C \times InhR \times EF \times ED}{{PEF \times BW \times AT_{nc \left( c \right)} }}$$

6 $$ADD_{der} = \frac{C \times SA \times AF \times ABF \times EF \times ED}{{BW \times AT_{nc \left( c \right)} }} \times 10^{ - 6}$$

Table S3 describes detailed information about the units, values, parameters, and receptors considered in the previous equations.

#### Non-carcinogenic risk

Hazard quotient (HQ) and hazard index (HI) were used to estimate the potential non-carcinogenic risk in the examined metal(loid)s (As, Cd, Cr, Cu, Mn, Ni, Pb, and Zn), applying the following equations (Schiavo et al., 2023a):

8 $$HI = \sum HQ_{i}$$

where *RfD*_*i*_ (mg kg^−1^ day^−1^) is the specific reference dose for each metal(loid) in the various exposure pathways, i.e., ingestion, inhalation, and dermal contact (Table S4). The non-carcinogenic risk is considered negligible if the HI value is less than 1. However, if the HI value is equal to or greater than 1, the non-carcinogenic risk is considered harmful to the health of adults and children (Dat et al., 2021).

#### Carcinogenic risk

The carcinogenic risk (CR) is reported for four metal(loid)s, As, Cr, Ni, and Pb, which are classified by the International Agency for Research on Cancer (IARC) as carcinogenic elements of group 1 (Cogliano et al., 2011). The CR and total CR (TCR) are calculated as follows:

9 $$CR = ADD_{i} \times SF$$

10 $$TCR = \sum CR_{i}$$

where *SF* (kg mg^−1^ day^−1^) is the carcinogenic slope factor (USEPA, 2010). A complete list of SF values for As, Cr, Ni, and Pb in three different pathways (ingestion, inhalation, and dermal contact) is shown in Table S4. For TCR values > 1 × 10^–4^ the risk is considered intolerable, while if TCR < 1 × 10^–6^, the risk is considered acceptable (Dat et al., 2021).

### Lung bioaccessibility test

Lung bioaccessibility test was performed in all ashfall samples using two synthetic lung fluids, GS and ALF, which mimic the extracellular (i.e., interstitial fluid) and intracellular (i.e., alveolar macrophage region) lung conditions, respectively (Meza-Figueroa et al., 2020). Table S5 shows the chemical composition of SLF used in this work.

The preparation of pulmonary solutions involved various steps: (1) the chemical agents, described in Table S4, were mixed in a specific order; (2) 0.1 g of volcanic ashfall was added to 10 mL of lung solutions, (GS and ALF) in a sterile plastic tube. According to Tomašek et al. (2021), the solid-to-liquid (S/L) ratio of 1/100 is a satisfactory option for volcanic environmental matrix to obtain a quality estimate of bioaccessibility; (3) the mixed solutions were incubated for 24 h at a temperature of 37 °C with a setting of 40 rpm; and finally, iv) the solutions were filtered with a 0.2 μm membrane (PVDF) and stored at 4 °C for future analytical sessions.

The extracted solutions of GS and ALF were measured by ICAP Qc (Thermo Fisher, USA) in the ICP-MS laboratory at the IGF-UNAM. SRM (NIST 2709A) and samples were prepared in duplicate, including a procedural blank for quality control of the analysis. Bioaccesible fraction (%Bio), expressed as a percentage, was calculated according to Boim et al. (2021) using the following formula:

11 $$\% Bio = \frac{{C_{bio} }}{{C_{tot} }} \times 100$$

where *C*_*bio*_ is the bioaccessible concentration of metal(loid)s in GS and ALF solutions (mg kg^−1^), and *C*_*tot*_ is the metal(loid) concentration (mg kg^−1^) of ashfall samples analyzed by ICP-MS (see Sect. "Sample collection and analysis").

### Statistical analysis

Statistical analysis, including graphical representations, were performed using XLSTAT and Python software (version 3.7), in order to interpret Popocatépetl volcanic ashfall data. Descriptive statistic parameters were implemented, including the evaluation of distribution degree, which was carried out by skewness calculation.

## Results and discussion

### Metal(loid) concentration in ashfall deposit

**Fig. 2:**
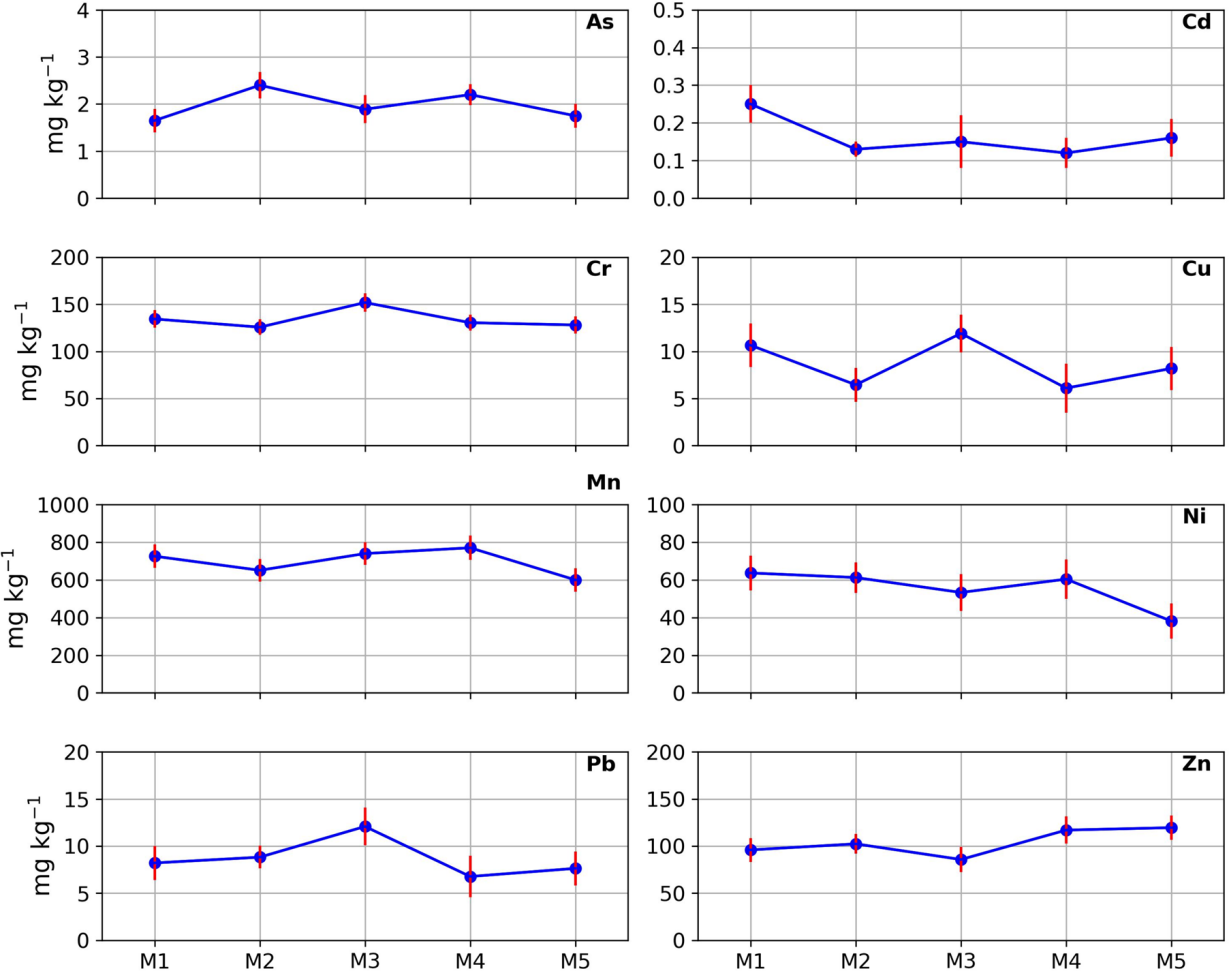
Concentration (mg kg^−1^) of metal(loid)s (As, Cd, Cr, Cu, Mn, Ni, Pb, and Zn) in ashfall deposits from Popocatépetl volcano, Mexico

The total concentration of eight metal(loid)s in ashfall deposits from Popocatépetl volcano and statistical characterizations, as well as their average concentration in local volcanic soil, are presented in Table 1 and Fig. 2. The mean concentrations of As, Cd, Cr, Cu, Mn, Ni, Pb, and Zn in ashfall deposits from Popocatépetl volcano were 1.98 ± 0.28 mg kg^−1^, 0.17 ± 0.05 mg kg^−1^, 134.09 ± 9.3 mg kg^−1^, 8.66 ± 2.3 mg kg^−1^, 697.33 ± 62.6 mg kg^−1^, 55.35 ± 9.3 mg kg^−1^, 8.78 ± 1.8 mg kg^−1^, and 104.10 ± 12.7 mg kg^−1^, respectively, with the following decreasing trend of Mn > Cr > Zn > Ni > Pb > Cu > As > Cd. The skewness coefficient (SC) of As, Cu, Mn, and Zn was close to 0, reflecting a quasi-normal distribution of the data. In contrast, other studied metal(loid)s, like Cd, Cr, Ni, and Pb, present a SC close to or greater than 1, corresponding to a lack of symmetry in data distribution. Positive skewness (Cd, Cr, and Pb), shows a right-skewed distribution, while negative skewness (Ni) describes a left-skewed distribution. The metal(loid) concentration in the different ash samples shows a very low variation, an expected result considering their common natural source, i.e., volcanic emissions. The coefficient of variation (CV) for each metal(loid) was 14.20% (As), 28.53% (Cd), 6.96% (Cr), 26.42% (Cu), 8.98% (Mn), 16.8% (Ni), 20.91% (Pb), and 12.24% (Zn). According to Yongming et al. (2008), a relatively low CV (< 50%) suggests a natural origin of metal(loid)s, while a relatively high CV (> 90%) reflects an anthropogenic source of metal(loid)s. Instead, intermediate CV values, between 50 and 90%, could suggest a combined natural and anthropogenic origin (Schiavo et al., 2021). Compared with metal(loid) concentrations reported in the local soil background, As, Cr, and Zn in the ashfall are enriched by 1.28, 1.39, and 1.28 times, respectively. On the contrary, ashfall metal(loid)s such as Cd (0.04), Cu (0.38), Mn (0.73), Ni (0.63), and Pb (0.15) showed an opposite trend compared to the background, with values less than 1. As reported in the article by Cruz-Sánchez et al. (2021), which compares the concentrations of certain metals from different eruptions, the volcanic ash of Popocatépetl contains high concentrations of Cr and Zn, and relatively lower concentrations of elements such as Pb, Ni and Cu. Furthermore, considering the concentrations present in the SRM-2709A (Table 1) agricultural contaminated soil, the average concentration of Mn in the ashfall was slightly higher, Cr and Zn showed similar concentrations, whereas other reported metal(loid)s, like As, Cd, Cu, Ni, and Pb, exhibited lower concentrations compared to the reference material.

In Table 2, a comparative descriptive analysis of the metal(loid) concentrations in ashfall deposits from Popocatépetl and other volcanoes worldwide is presented. The average concentration of As in our study (1.98 mg kg^−1^) is notably lower than those reported from Tolbachik, Russia (3.4 mg kg^−1^), Merapi and Sinabung, Indonesia (8.9 and 4.5 mg kg^−1^, respectively), Copahue, Chile (7.6 mg kg^−1^), Puna, Argentina (5.2 mg kg^−1^), and Ruapehu, New Zealand (6.3 mg kg^−1^), but higher than Kizimen, Russia (1 mg kg^−1^). In Copahue, Chile, the Cd mean concentration (0.41 mg kg^−1^) was slightly similar to the values reported in this work (0.17 mg kg^−1^) but significantly lower compared to Merapi, Indonesia (1.3 mg kg^−1^, respectively) and Ruapehu, New Zealand (1 mg kg^−1^). The Cr and Ni concentrations in our samples (134.09 and 55.35 mg kg^−1^, respectively) were 1.5–36.2 times higher than those reported in all the volcanoes considered in this comparison (average concentration of 17.5 and 10.9 mg kg^−1^, respectively). On the other hand, the mean Cu concentrations from Popocatépetl volcano (8.66 mg kg^−1^) were found lower compared to all selected worldwide volcanoes (average concentration of 64.9 mg kg^−1^), except for Merapi volcano, Indonesia (7.4 mg kg^−1^). The concentration of Mn (697.33 mg kg^−1^) and Zn (104.10 mg kg^−1^) was higher in Merapi, Indonesia (1310 and 160.6 mg kg^−1^, respectively) and Sinabung, Indonesia (1800 and 109 mg kg^−1^, respectively), but lower than Mt. St. Helens, USA (501 and 56 mg kg^−1^, respectively), Puna, Argentina (600 and 39.8 mg kg^−1^, respectively), and Ruapehu, New Zealand (34.7 and 25.8 mg kg^−1^, respectively). Similar values of Zn in ashfall were found in Tolbachik, Russia (103 mg kg^−1^) and Copahue, Chile (102.6 mg kg^−1^). Finally, the Pb concentration (8.78 mg kg^−1^) were found to be higher compared with ashfall samples from Tolbachik and Kizimen, Russia (7.2 and 4.4 mg kg^−1^), and Ruapehu, New Zealand (2.7 mg kg^−1^), but significantly lower than Mt. St. Helens, USA (19 mg kg^−1^) and Copahue, Chile (14.3 mg kg^−1^).

**Table 2:** Comparison of average metal(loid) (mg kg^−1^) concentrations in Popocatépetl ashfall with other volcanoes worldwide

Study area	As	Cd	Cr	Cu	Mn	Ni	Pb	Zn
Popocatépetl, Mexico^a^	1.98	0.17	134.09	8.66	697.33	55.35	8.70	104.10
Mt. St. Helens, USA^b^	nr	nr	15	42	501	14	19	56
Tolbachik, Russia^c^	3.4	nr	3.7	242	nr	9	7.2	103
Kizimen, Russia^c^	1	nr	6.7	23.1	nr	5.6	4.4	48.2
Merapi, Indonesia^d^	8.95	1.29	3.88	7.41	1310	2.77	10.14	160.60
Sinabung, Indonesia^e^	4.48	nr	11.80	nr	1800	nr	nr	109
Copahue, Chile^f^	7.60	0.41	89.70	52.63	1082	29.17	14.26	102.60
Puna, Argentina^g^	5.20	nr	4.5	nr	600	nr	nr	39.8
Ruapehu, New Zealand^h^	6.3	1	7.6	22.2	34.7	4.8	2.7	25.8

*Nr* not reported

^a^This study

^b^Taylor and Lichte (1980)

^c^Ermolin et al. (2021)

^d^Salamah and Wahyuni (2018)

^e^Kusmartini et al. (2017)

^f^Ruggieri et al. (2011)

^g^Ruggieri et al. (2010)

^h^Cronin et al. (1998)

The concentrations of metals emitted by volcanoes depend significantly on the geodynamic context and origin of the magma. Arc volcanoes typically emit a greater flux of certain metals, like Cu, Pb, and Zn, that partition into vapor from silicate melt (Hinkley et al., 1999). On the other hand, the metal flux from intraplate (i.e. hotspot) volcanoes is generated from the oxidation of liquid sulfide during degassing and the rise of magma. Additionally, the differences in metal flux are controlled by magma differentiation and decompression, as well as interaction between melts and sulfides. The composition of the magma plays a crucial role in the emissions and the quantity of gases and trace elements released into the environment. A volcano emits volatile metals in the aerosol phase and their concentrations are closely related to deposition rate, leaching, geochemical cycle, and mineralization (Edmonds & Mather, 2017). The metal emission fluxes depend on the eruptive style and the alternation of active and quiescent periods, characterized by eruption and passive degassing, respectively (Edmonds et al., 2022).

### Mineral composition of ashfall deposit

The spectral XRD analysis (Fig. S2A–E) revealed the presence of several mineralogical phases typical of magmatic environments (Table 3). Ashfall samples from Popocatépetl contain silicate minerals group (e.g., inosilicate and tectosilicate), and Si-Fe oxides in the following order of relative average abundances: andesine (81.1%) > diopside (7.3%) > magnetite (6.1%) > cristobalite (3.4%) > augite (2.2%). The recognized minerals have different characteristics and chemical composition: i) andesine ((Ca, Na)(Al, Si)_4_O_8_) is a solid solution of plagioclase feldspar that represents a tectosilicate group; ii) diopside (Ca(Mg, Mn, Fe)Si_2_O_6_) and augite ((Ca, Mg, Fe)_2_(Si, Al)_2_O_6_)) are classified as clinopyroxene, which constitute an inosilicate group; and, iii) cristobalite (SiO_2_) and magnetite (Fe_3_O_4_) are a Si- and Fe-oxides, respectively. Magnetite is a mineralogical phase present in the subgroup of spinel with a general chemical formula AB_2_O_4_ (A and B are element such as Mn, Cr, Fe, Mg, and Al). Instead, cristobalite is a polymorph with the same chemical formula of quartz (SiO_2_) but different crystalline structure. The formation of cristobalite depends by devitrification and vapor-phase crystallization from andesitic magma (Baxter et al., 1999), and it’s a product of the partial collapse of the lava dome during eruption.

**Table 3:** Mineralogical phases in ashfall samples from Popocatépetl volcano

	Andesine	Cristobalite	Diopside	Magnetite	Augite
M1	81.9	3.4	6.9	1.2	6.7
M2	80.6	3.2	7.3	7.6	1.2
M3	77.6	4.2	8.3	8.8	1
M4	82.9	2.7	6.8	6.7	0.9
M5	82.3	3.4	7.2	6	1

Values are expressed in %

The minerals identified with XRD are representative of an andesitic/dacitic magma, confirming the petrological investigation carried out by Witter et al. (2005). The composition of Popocatépetl rocks is a mixture of basaltic/andesitic and dacitic magma, including minerals like olivine, plagioclase, pyroxene, and Fe-Ti oxides (Schaaf et al., 2005; Witter et al., 2005). Heavy metals in ashfall deposit are present in trace amounts which in many cases are difficult to detect with XRD due to the sensitivity of the instrument, as well as their abundance in the mineralogical phase and the degree of crystallinity.

### Volcanic ashfall particle distribution

**Fig. 3:**
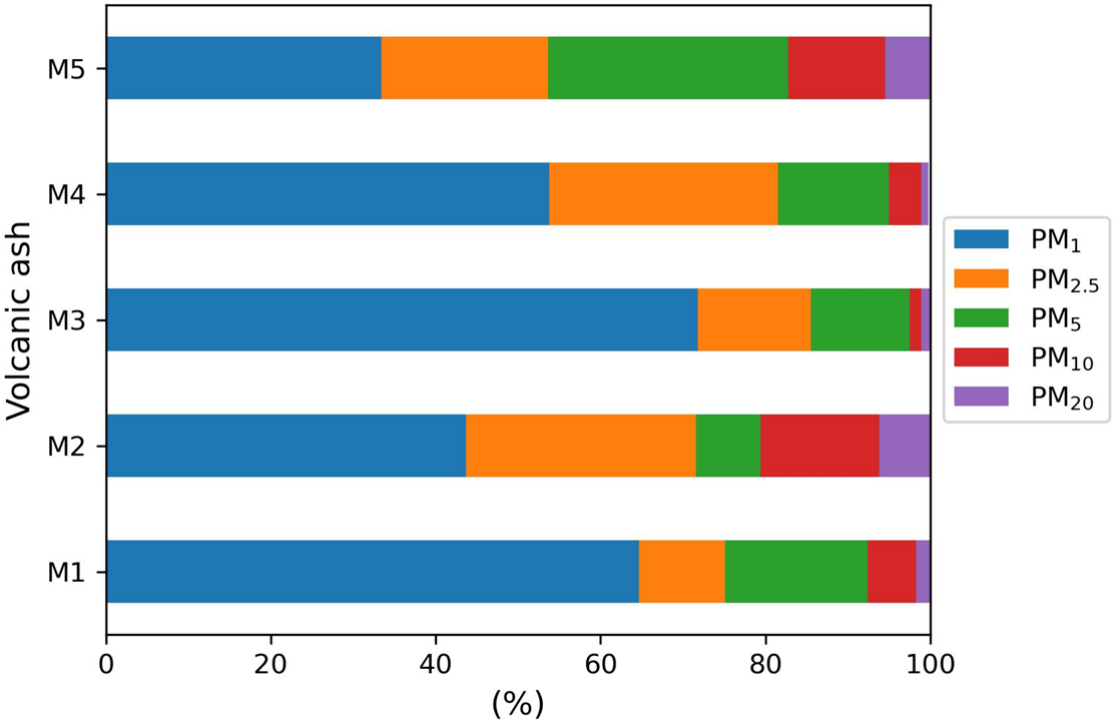
Analysis of grain-size distribution of ashfall particles realized using SEM–EDS. Aerodynamic diameter values of volcanic ash are expressed in percentage (%)

Particle size distribution of five ashfall samples from Popocatépetl was obtained by SEM using PMS software (Fig. 3). The size of particles was classified as follows: PM_20_ (< 20 μm), PM_10_ (< 10 μm), PM_5_ (< 5 μm), PM_2.5_ (< 2.5 μm), and PM_1_ (< 1 μm). On average, the particles present in the ashfall are found in a range between 0.21 and 42 μm. All samples (M1–M5) show a variable distribution considering the different sizes and high percentage (> 50%) of fine and extremaly fine, PM_2.5_ and PM_1_, respectively, particles. The highest concentration of PM_1_ was detected in samples M1, M3, and M4, with an approximate percentage of 61, 72, and 54%, respectively. On the contrary, in M2 and M5 sample, the concentration of sub-micrometric particles was found less than 50%, with a value of about 42 and 38%, respectively. Coarse particles (PM_10_ and PM_20_) are present in small quantities, with an average percentage from 2.5 to 20.5%. The examined ash particles are characterized by the ability to be resuspended and transported over long distances in the atmosphere. PM_2.5_ and PM_1_, classified as dangerous for human health, can easily penetrate in the deep part of the lungs, interact with the alveolar region, pass through the cellular barrier, and reach the bloodstream. Once in the bloodstream, particles have the ability to disperse throughout the body and reach various organs and tissues, promoting cardiovascular and neuroinflammation with strong health effects, including a cognitive decline and poor quality life. Exposure to PM_2.5_ and PM_1_ is also related to increased likelihood of developing different forms of cancer (Zhang et al., 2024; NASEM, 2024).

Generally, as reported by some studies (Horwell & Baxter, 2006; Horwell, 2007), volcanic ash is composed of fine to extremely fine particles. The particle size fraction is directly related to the eruptive style of the volcano, the fragmentation degree of magma, and the amount of dissolved gases, among others factors. Farther important mechanism involving PM_1_ and ultrafine (< 100 nm) particles in volcanic ash is the aggregation process (Paredes-Mariño et al., 2019). As reported in the literature (Trejos et al., 2021), ultrafine particles tend to aggregate into homogeneous clusters. The degree of aggregation influences the behavior of the particles once in contact with biological fluids. Schiavo et al. (2023b) demonstrated that fine particles serve as carrier for ultrafine particle exposure. In contact with the acidic fluid of the alveolar macrophages (deep part of the lungs) fine particles tend to disaggregate, dispersing ultrafine particles into the human body. This can lead to an inflammatory response and death of the macrophage inside the alveolar region. These events are related to chronic respiratory conditions and have long-term health implications. Additional effects include oxidative stress promoting the dysregulation of immune responses, increasing susceptibility to respiratory infections and exacerbating chronic conditions like asthma (Ling and van Eeden 2009).

### Environmental assessment

**Fig. 4:**
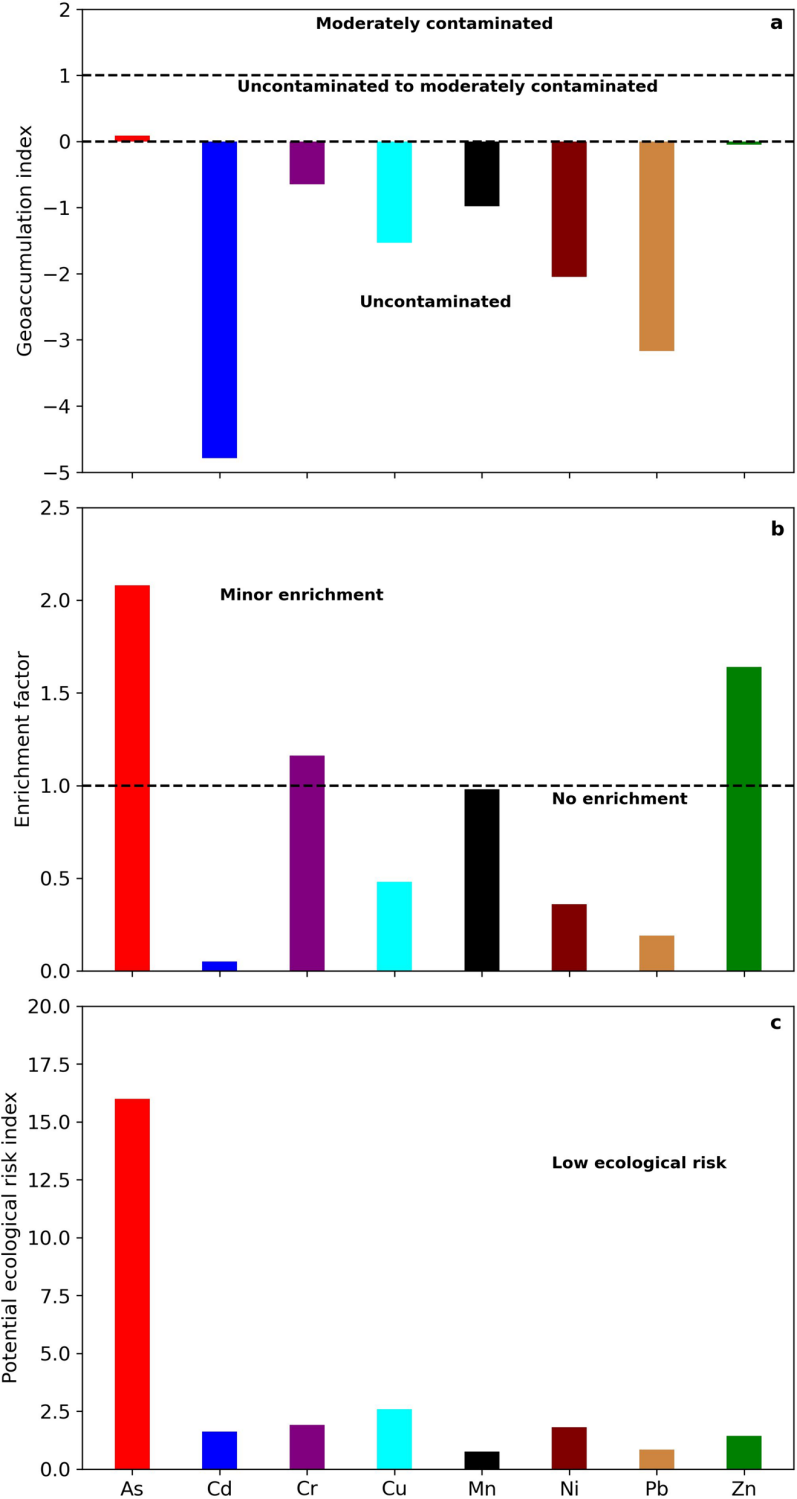
**a** Geoaccumulation index (Igeo), **b** Enrichment factor (EF), and **c** Potential ecological risk index (RI)

In the present work, I_geo_ was used to evaluate anomalous levels of metal(loid)s compared to baseline concentration (i.e., Popocatépetl soil). The I_geo_ results of metal(loid)s in ashfall samples are presented in Fig. 4a. I_geo_ mean values for As, Cd, Cr, Cu, Mn, Ni, Pb, and Zn were 0.09, − 4.79, − 0.65, − 1.53, − 0.98, − 2.05, − 3.17, and − 0.05, respectively. Comparing to background level, all studied metal(loid)s, except As, are classified as uncontaminated (I_geo_ ≤ 1). The maximum Igeo value was observed for As, which is classified between uncontaminated to moderate contaminated (0 < Igeo ≤ 1).

The EF is a normalization technique used to assess the impact of toxic metal(loid)s in different fractions. EF calculations report the following values in descending order: As (2.08) > Zn (1.64) > Cr (1.16) > Mn (0.98) > Cu (0.48) > Ni (0.36) > Pb (0.19) > Cd (0.05). Considering the mean EF values, As, Zn, and Cr were classified with a minor enrichment (1 ≤ EF ≤ 3), while other metal(loid)s like Mn, Cu, Ni, Pb, and Cd are set in the no enrichment (EF < 1) category. As can be seen in Fig. 4b, the maximum level of EF was found for As with an approximate increase of 1.5% compared to the background and a similar distribution pattern as I_geo_. Considering the results of I_geo_ and EF geochemical indices, among the metal(loid)s studied in this work, As is slightly enriched compared to the baseline concentration.

Figure 4c shows the potential ecological risk assessment (RI) result of metal(loid)s in ashfall samples. The mean RI of metal(loid)s in ashfall increases following the order of Mn (0.76), Pb (0.84), Zn (1.44), Cd (1.63), Ni (1.81), Cr (1.91), Cu (2.59), and As (16). RI values are lower than 40, indicating low environmental ecological risk. As in the case of Igeo and EF, the highest level was reported for As, which shows values on average 10 times higher compared to other metal(loid)s. Anyway, volcanic soils, enriched with essential elements and minerals, are considered suitable for the proliferation of vegetation (Lai et al., 2022). Recent studies (Pickarski et al., 2023) show that the vegetation adapts to volcanic conditions after long periods (i.e. years). As reported by Baillie et al. (2018) the main limitation for the development of vegetation is the presence of toxic gases, especially sulfur compounds, and the consequent formation of acid rain.

### Health risk assessment

In this study, human health risk from three exposure pathways (ingestion, inhalation, and dermal absorption) to eight metal(loid)s in volcanic ashfall were characterized using non-carcinogenic and carcinogenic risk models. The ADD values of metal(loid)s decrease in the following order of Mn > Cr > Zn > Ni > Pb > Cu > As > Cd for children and adults through each pathway (Table S6), which is the same order considering the average metal(loid)s concentration in ashfall (Sect. "Metal(loid) concentration in ashfall deposit"). Assessment of ADD for children and adults indicated that ingestion was the most critical route of exposure compared to dermal absorption and inhalation.

The estimation of non-carcinogenic (HQ) human health risk assessment is presented in Table 4. Results show significant differences between the exposure pathways: HQ_ing_ > HQ_der_ > HQ_inh_. Oral ingestion of As, Cd, Cr, Cu, Mn, Ni, Pb, and Zn was recognized as the main exposure route for adults and children. The risk values for inhalation and dermal contact were found to be lower than ingestion, with a difference between 1 and 6 orders of magnitude. Cr was found to produce the highest HQ via ingestion for children (5.71E−01) and adults (6.12E−02). Similarly, Mn was found to be the highest risk element via inhalation and dermal absorption for children (4.98E−03 and 2.60E−02, respectively) and adults (2.81E−03 and 3.97E-03, respectively). However, considering different routes of exposure, no adverse effects were found in the target population, with HQ values < 1. The total non-carcinogenic (HI) health risks for children were 8.49E−02 (As), 2.31E−03 (Cd), 5.73E−01 (Cr), 2.78E−03 (Cu), 4.02E−01 (Mn), 3.55E−02 (Ni), 7.97E−02 (Pb), and 4.45E−03 (Zn). On the other hand, for adults the HI were 9.12E−03 (As), 2.61E−04 (Cd), 6.22E−02 (Cr), 2.98E−04 (Cu), 4.66E−02 (Mn), 3.81E−03 (Ni), 8.55E−03 (Pb), and 4.77E−04 (Zn). The HI values decreased in the following order for children and adults: Cr > Mn > As > Pb > Ni > Zn > Cu > Cd. All metal(loid)s in ashfall obtained in this work had HI values less than 1, which indicates no carcinogenic risk. Elements such as Cr and Mn, even not reaching the limit value of 1, reported the highest HI values, 0.57 and 0.40 respectively. Moreover, HI values of metal(loid)s for children were almost an order of magnitude higher than those for adults. Several authors (Han et al., 2020; Schiavo et al., 2023a) reported a correlation between pollution and chronic diseases in a target population. Children, due to their lung function, hand-to-mouth activity, body weight, and immune system still developing, are more vulnerable and sensitive to atmospheric pollution. Considering cumulative risk, assessed by calculating the sum of HQ values for selected metal(loid)s, the HI was up to 1.19 for children and 0.13 for adults. Under these conditions, a significant non-carcinogenic health risk was detected for the population residing in the area affected by volcanic ash.

**Table 4:** Non-carcinogenic risk (HQ) of metal(loid)s in volcanic ashfall deposit for children and adults

Metal(loid)	HQ_ing_		HQ_inh_		HQ_der_		HI	
	Children	Adults	Children	Adults	Children	Adults	Children	Adults
As	8.43E−02	9.03E−03	1.41E−06	7.97E−07	5.76E−04	8.79E−05	8.49E−02	9.12E−03
Cd	2.07E−03	2.22E−04	5.79E−06	3.26E−06	2.32E−04	3.54E−05	2.31E−03	2.61E−04
Cr	5.71E−01	6.12E−02	1.67E−03	9.45E−04	2.46E−04	3.76E−05	5.73E−01	6.22E−02
Cu	2.77E−03	2.97E−04	2.58E−07	1.45E−07	7.75E−06	1.18E−06	2.78E−03	2.98E−04
Mn	3.71E−01	3.98E−02	4.98E−03	2.81E−03	2.60E−02	3.97E−03	4.02E−01	4.66E−02
Ni	3.54E−02	3.79E−03	3.66E−06	2.07E−06	9.91E−05	1.51E−05	3.55E−02	3.81E−03
Pb	7.95E−02	8.51E−03	8.83E−07	4.98E−07	2.23E−04	3.40E−05	7.97E−02	8.55E−03
Zn	4.44E−03	4.75E−04	1.06E−09	5.99E−10	1.24E−05	1.90E−06	4.45E−03	4.77E−04
Cumulative HI							1.19	0.13

The CR of As, Cd, Cr, Ni, and Pb via three exposure pathways are show in Table 5. Certain substances are considered carcinogenic according to the International Agency for Research on Cancer (IARC). Such elements as As, Cd, Cr, and Ni are inserted in group 1 of substances carcinogenic to humans. Instead, Pb is included in group 2B of possibly cancerogenic substances for humans. As described for non-carcinogenic risk, the highest CR was found during ingestion exposure, followed by dermal contact and inhalation. The TCR values for children were 3.27E−06 (As), 3.13E−11 (Cd), 7.36E−05 (Cr), 1.42E−09 (Ni), and 8.11E−08 (Pb). Instead, considering adult receptors, the TCR were 4.11E-06 (As), 2.06E−10 (Cd), 9.30E−05 (Cr), 9.37E−09 (Ni), and 1.01E−07 (Pb). The mean TCR of metal(loid)s in ashfall deposits were in the order of Cr > As > Pb > Ni > Cd for children and adults. Compared to the HI results, similar TCR value were found among children and adults. Metal(loid)s like As, Cr, and Ni report comparable values between children (3.25E−06, 7.35E−05, and 1.42E−09, respectively) and adults (4.06E−06, 9.18E−05, and 9.37E−09, respectively). On the other hand, considering TCR of Cd and Pb, adults’ values were approximately an order of magnitude higher than children’s. All metal(loid)s, except for Cr, showed an acceptable TCR. Cr is the only metal(loid) that returns a TCR value cataloged as tolerable. Likewise, the cumulative TCR values of selected metal(loid)s was found higher than 1.0E-06, indicating a moderate carcinogenic health risk for children and adults in the study area.

**Table 5:** Carcinogenic risk (CR) of specific metal(loid)s in volcanic ashfall deposit for children and adults

Metal(loid)	CR_ing_		CR_inh_		CR_derm_		TCR	
	Children	Adults	Children	Adults	Children	Adults	Children	Adults
As	3.25E−06	4.06E−06	9.15E−10	6.02E−09	2.22E−08	3.96E−08	3.27E−06	4.11E−06
Cd	–	–	3.13E−11	2.06E−10	–	–	3.13E−11	2.06E−10
Cr	7.35E−05	9.18E−05	1.68E−07	1.11E−06	–	–	7.36E−05	9.30E−05
Ni	–	–	1.42E−09	9.37E−09	–	–	1.42E−09	9.37E−09
Pb	8.11E−08	1.01E−07	1.12E−11	7.36E−11	–	–	8.11E−08	1.01E−07
Cumulative TCR							7.70E−05	9.72E−05

The equations developed by the USEPA are widely used in the literature but may underestimate health risks. Different factors are not considered in the USEPA model, including the nature and reactivity of the particle, element oxidation state, particle shape and circularity, and mineralogical phase. Especially the oxidation state of certain heavy metals such as As, Cd, Cu, and Cr can generate important harmful and toxic effects (Jaishankar et al., 2014), inhibiting the action of free radicals and cell growth. Further toxicological studies, including bioaccessibility assessment, as well as in vitro and in vivo tests, are necessary to increase our knowledge about the effects of volcanic ash on health.

### Lung bioaccessibility

**Fig. 5:**
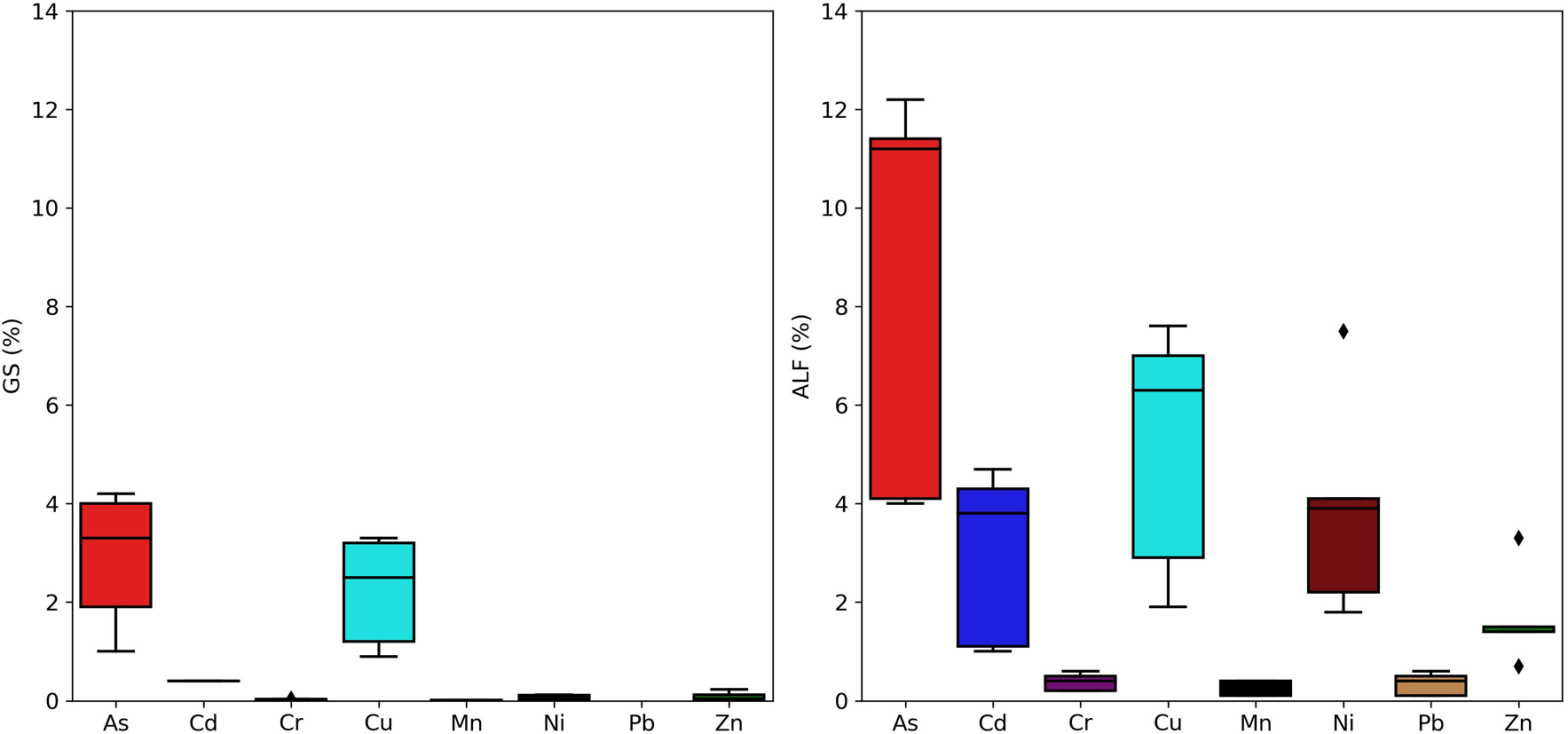
Lung bioaccessibility (%) in GS and ALF of several metal(loid)s from ashfall deposits collected at the Popocatépetl volcano

The lung bioaccessibility of metal(loid)s associated with volcanic ashfall deposits in GS and ALF is presented in Table 6. Figure 5 shows boxplots with the relative percentages (%) of the bioaccessible fraction of metal(loid)s in the analyzed ashfall samples. The relative percentages are influenced by the nature of the in vitro lung solutions. In this case, the type of digestion is considered incomplete, simulating the conditions inside the human lung.

**Table 6:** Lung bioaccessibility, Gamble solution (GS) and Artificial Lysosomal Fluid (ALF), of metal(loid)s in Popocatépetl volcanic ashfall

	As	Cd	Cr	Cu	Mn	Ni	Pb	Zn
*GS*								
2709a	1.79	0.34	0.06	1.54	0.11	0.10	< LOD	0.10
M1	4.20	0.36	0.04	3.21	0.01	0.11	< LOD	0.23
M2	3.28	< LOD	0.03	0.90	0	0.03	< LOD	0.08
M3	1.94	< LOD	0.03	3.28	0.01	0.09	< LOD	0.01
M4	1	< LOD	0.01	1.20	0	0.03	< LOD	< LOD
M5	4.04	< LOD	0.03	2.54	0.01	0.12	< LOD	0.01
*ALF*								
2709a	12.47	10.95	2.59	7.75	8.92	7.73	2.45	4.43
M1	11.21	1	0.19	7.57	0.14	1.77	0.10	3.33
M2	11.36	3.77	0.41	6.25	0.18	3.92	0.47	1.37
M3	4.12	1.07	0.16	2.91	0.07	2.24	0.14	0.73
M4	3.99	4.67	0.47	1.91	0.38	4.15	0.35	1.46
M5	12.18	4.31	0.61	7	0.35	7.49	0.63	1.40

Values are expressed in %

The GS bioaccessibility of As, Cd, Cr, Cu, Mn, Ni, and Zn in ashfall varied from 1 to 4.20% (mean of 2.89%), 0.4%, 0.01 to 0.04% (mean of 0.03%), 0.90 to 3.28% (mean of 2.22%), 0 to 0.01% (mean of 0.01%), 0.03 to 0.12% (mean of 0.08%), and 0.01 to 0.23% (mean of 0.08%), respectively. On the other hand, considering As, Cd, Cr, Cu, Mn, Ni, Pb, and Zn in ashfall, ALF bioaccessibility varied from 3.99 to 12.18% (mean of 8.57%), 1 to 4.67% (mean of 2.96%), 0.16 to 0.61% (mean of 0.36%), 1.91 to 7.57% (mean of 5.12%), 0.14 to 0.38% (mean of 0.22%), 1.77 to 7.45% (mean of 3.19%), 0.10 to 0.63% (mean of 0.34%), and 0.73 to 3.33% (mean of 1.66%), respectively. The bioaccessible concentration of Pb in Gamble lung solution was found to be very low, with all samples below the detection limit. Similar result was observed for Cd, with only one sample (M1) reporting a bioaccessibility value in the GS. The average metal(loid)s bioaccessibility in Gamble and ALF solutions followed the decreasing order of As > Cu > Cd > Ni = Zn > Cr > Mn and As > Cu > Ni > Cd > Zn > Cr > Pb > Mn, respectively. The bioaccessible fraction of metal(loid)s in Gamble is reported lower than ALF solution. This trend is described in several works available in the literature (e.g., Expósito et al., 2021; Hernández-Pellón et al., 2018), which is attributed to the acidic conditions present in the alveolar region compared to the neutral pH of the extracellular area. The bioaccessibility values of GS and ALF were also compared with SRM 2709a San Joaquin agricultural soil. In GS, the mean bioaccessible fraction of Cd, Cr, and Mn did not exceed the reference material values, which shows higher bioaccessibility. Instead, the mean GS bioaccessibility of As and Cu exceeded 1.6 and 1.5 times the values of the reference material, respectively. Considering the ALF solution, the bioaccessible fraction of metal(loid)s in all studied samples did not exceed the SRM 2709a values. Volcanic ash resulting slightly biosolubles or insoluble (Horwell et al., 2003) compared to anthropogenic materials, which are typically characterized by easily dissolvable complexes such as oxides, carbonates, and chlorides. Meza-Figueroa et al. (2020) reported lung bioaccessibility of several heavy metals in urban dust samples. The bioaccessible values ranged from 0.01 to 79.5% in GS and 2 to 100% in ALF. Furthermore, Hernández-Pellón et al. (2018) compared the pulmonary bioaccessibility between urban and industrial areas. The bioaccesible fractions were higher in the industrial area (from 4 to 47.3% and 23.4 to 89% in GS and ALF, respectively) compared to the urban area (from 1.3 to 23% and 12.2 to 77.6% in GS and ALF, respectively). The mechanism of bioaccessibility is controlled by particle morphology, size distribution, and chemical composition of compounds (Schiavo et al., 2021). Small-sized circular particles are easily dissolved and absorbed by the human body. Volcanic materials, mainly composed of crystalline silicate minerals and angular particles, are considered biodurable, bioreactive, and poorly bioaccessible once in contact with body fluids (Plumlee & Ziegler, 2007). However, solubility may change depending on the weathering leaching process, mineral alterations, and the presence of non-polar hydrophobic elements. Surface volcanic ash particles are also characterized by a chronic bioreactivity, which plays a crucial role in modifying body fluid parameters like redox species, electrolyte concentrations, and pH. The insoluble particles of volcanic ash remain in the lungs, slowly reacting with fluids. The slow reaction and release of chemical species with lung fluids leads to the generation of reactive oxygen species (ROS). ROS formation is directly related to tissue damage, oxidative stress, and consequent health risks from inhalation exposure to volcanic ash (Plumlee & Ziegler, 2007).

Biodurable and poorly bioaccessible volcanic ash particles persist in the lungs as they do not easily dissolve in pulmonary fluids, remaining intact for longer periods and making their elimination by the natural cleaning mechanisms of the lung more difficult. Consequently, their physical presence can cause potentially chronic irritation or inflammation in the tissue lungs, which can be particularly dangerous for vulnerable populations such as the elderly, children, and those with pre-existing conditions such asthma or some viral lung infection. Additionally, alveolar macrophages residing in the lungs can phagocytose volcanic ash particles smaller than 2.5 microns. This process is part of the mechanisms of innate immune response to eliminate inhaled foreign particles. Phagocytosis can trigger a series of intracellular events including the production of cellular damage, free radicals, and even cell death, as well as the release of pro-inflammatory cytokines. This cycle of damage and inflammation can result in a persistent state of oxidative stress in lung tissues, which can lead to long-term lung damage, exacerbation of pre-existing conditions, and increased susceptibility to infections. These processes can significantly contribute to the adverse respiratory health effects related with the inhalation of biodurable and poorly bioaccessible volcanic ash. Further studies on the interaction between volcanic ash, possible exposure routes, and biological fluids must be conducted in order to better understand the health impacts in short and long-term. This information will be crucial for developing public policies and mitigation strategies to protect vulnerable population exposed to volcanic ash, particularly in children due to their developing respiratory, neurological, and immune system.

## Limitations of study

This study focused on a deterministic model for risk estimation and in vitro tests to evaluate bioaccessibility and biopersistence of respirable particles. However, in this case, it was no possible to develop biomarker tests for human exposure or effect. Furthermore, we provided general information about the potential risks of bio-persistent minerals in breathable sizes. This information can help volcano risk decision-makers adopt measures to reduce the population's exposure. Our findings highlight the relevance of particle size distribution in ash fall and bioaccessibility in vitro tests for a more reliable health risk assessment. Further studies including biomarkers of exposure and effect are highly recommended.

## Conclusions

The present study analyzed concentration, particle size distribution, health risk assessment, and lung bioaccessibility in ashfall samples collected from the volcanic area of Popocatépetl. The primary metal(loid)s investigated were As, Cd, Cr, Cu, Mn, Ni, Pb, and Zn. All studied metal(loid)s, except for As, Cr, and Zn, were found in lower amounts compared to the natural soil background. The results of risk assessment through ingestion, inhalation, and dermal contact suggested an important non-carcinogenic risk. An unacceptable exposure risk (HQ = 1.2) was found in children considering the cumulative risk of studied metal(loid)s. Taking into consideration the different routes of exposure, the higher risk for children and adults was reported during ingestion, followed by dermal contact and inhalation. Additionally, a tolerable carcinogenic risk was registered in ashfall samples, with values between 1E-11 and 1E-05. Bioaccessibility in SLF of all examined metal(loid)s showed less variability between ashfall samples. An expected result considering volcanic emission source. Moreover, a low metal solubility (< 20%) in GS and ALF was reported for all metal(loid)s. The highest bioaccesible fractions was recorded in ALF solution for As and Cu with values of 12.2% and 7.6%, respectively. Volcanic ash is less bioaccessible compared to anthropogenic samples, which show a moderate (between 30 and 70%) to high (> 70%) trend. This result can be attributed to the mineralogical composition, surface characteristics, and chemical stability of volcanic materials, which are mainly formed by silicate minerals. Silicates exhibit higher stability and low solubility, rendering the compounds bioinsoluble and biodurable when in contact with biological fluids. Crystalline material are hazardous to health due to potential toxicity mechanisms like those causing silicosis or mesothelioma. The ashfall samples were also characterized by the presence of fine and extremely fine particles smaller than 2.5 and 1 μm, respectively, which are classified as highly inhalable and hazardous to health. Since urbanized areas are commonly close to active volcanoes, more research on the particle size distribution of volcanic ashes is strongly recommended.

Future research should focus on characterizing of the volcanic ash bioinsoluble fraction and assessing the potentially hazardous effect on human health, as well as to evaluating their gastrointestinal bioaccessibility and oxidative potential.

## Supplementary Information

Below is the link to the electronic supplementary material.

Supplementary file1 (DOCX 1273 KB)

## Data Availability

The data that support the findings of this study are available on request from the authors.

## References

[CR1] Aiuppa, A., Baker, D. R., & Webster, J. D. (2009). Halogens in volcanic systems. *Chemical Geology,**263*(1–4), 1–18. 10.1016/j.chemgeo.2008.10.005 10.1016/j.chemgeo.2008.10.005

[CR2] Amaral, A. F. S., Arruda, M., Cabral, S., & Rodrigues, A. S. (2008). Essential and non-essential trace metals in scalp hair of men chronically exposed to volcanogenic metals in the Azores, Portugal. *Environmental International,**34*(8), 1104–1108. 10.1016/j.envint.2008.03.013 10.1016/j.envint.2008.03.013 18485481

[CR3] Baillie, C.-K., Kaufholdt, D., Meinen, R., Hu, B., Rennenberg, H., Hansch, R., & Bloem, E. (2018). Surviving volcanic environments: Interaction of soil mineral content and plant element composition. *Frontiers in Environmental Science*. 10.3389/fenvs.2018.00052 10.3389/fenvs.2018.00052

[CR4] Barclay, J., Haynes, K., Houghton, B., & Johnston, D. (2015). *Social processes and volcanic risk reduction. The Encyclopedia of volcanoes* (2nd ed., pp. 1203–1214). Academic Press. 10.1016/B978-0-12-385938-9.00069-9

[CR5] Baxter, P. J., Bonadonna, C., Dupree, R., Hards, V. L., Kohn, S. C., Murphy, M. D., Nichols, A., Nicholson, R. A., Norton, G., Searl, A., & Sparks, R. S. J. (1999). Cristobalite in volcanic ash of the Soufrière Hills volcano, Monserrat, British West Indies. *Science,**283*(5405), 1142–1145. 10.1126/science.283.5405.1142 10024235 10.1126/science.283.5405.1142

[CR6] Boim, A. G. F., Patinha, C., Wragg, J., Cave, M., & Ferracciú-Avelloni, L. R. (2021). Respiratory bioaccessibility and solid phase partitioning of potentially harmful elements in urban environmental matrices. *Science of the Total Environment,**765*, 142791. 10.1016/j.scitotenv.2020.142791 33097248 10.1016/j.scitotenv.2020.142791

[CR7] Brown, S. K., Sparks, R. S. J., & Jenkins, S. F. (2015). *Global distribution of volcanic threat. Global volcanic hazards and risk* (pp. 359–370). Cambridge University Press. 10.1017/CBO9781316276273.025

[CR8] Cakmak, D., Perovic, V., Kresovic, M., Pavlovic, D., Pavlovic, M., Mitrovic, M., & Pavlovic, P. (2020). Sources and a health risk assessment of potentially toxic elements in dust at children’s playgrounds with artificial surfaces: A case study in belgrade. *Archive of Environmental Contamination and Toxicology,**78*, 190–205. 10.1007/s00244-019-00702-0 10.1007/s00244-019-00702-0 31901970

[CR9] Camarinho, R., Ventura García, P., Choi, H., & Rodrigues, A. (2021). Pulmonary oxidative stress and apoptosis in mice chronically exposed to hydrothermal volcanic emissions. *Environmental Science and Pollution Research,**28*, 35709–35716. 10.1007/s11356-021-13043-0 33675493 10.1007/s11356-021-13043-0

[CR10] Cogliano, V. J., Baan, R., Straif, K., Grosse, Y., Lauby-Secretan, B., El Ghissassi, F., Bouvard, V., Benbrahim-Tallaa, L., Guha, N., Freeman, C., & Galichet, L. (2011). Preventable exposures associated with human cancers. *Journal of the National Cancer Institute,**103*(24), 1827–1839. 10.1093/jnci/djr483 22158127 10.1093/jnci/djr483 PMC3243677

[CR11] Conte, G., Urrutia-Fucugauchi, J., Goguitchaichvili, A., Soler-Arechalde, A. M., Morton-Bermea, O., & Incoronato, A. (2004). Paleomagnetic study of lavas from the Popocatépetl volcanic region, Central Mexico. *International Geology Review,**46*(3), 210–225. 10.2747/0020-6814.46.3.210 10.2747/0020-6814.46.3.210

[CR12] Covey, J., Dominelli, L., Horwell, C. J., Rachmawati, L., Martin-del Pozzo, A. L., Armienta, M. A., Nugroho, F., & Ogawa, R. (2021). Carers’ perceptions of harm and the protective measures taken to safeguard children’s health against inhalation of volcanic ash: A comparative study across Indonesia, Japan and Mexico. *International Journal of Disaster Risk Reduction,**59*, 102194. 10.1016/j.ijdrr.2021.102194 10.1016/j.ijdrr.2021.102194

[CR13] Cronin, S. J., Hedley, M. J., Neall, V. E., & Smith, R. G. (1998). Agronomic impact of tephra fallout from the 1995 and 1996 Ruapehu Volcano eruptions, New Zealand. *Environmental Geology,**34*, 21–30. 10.1007/s002540050253 10.1007/s002540050253

[CR100] Cruz-Sánchez, M., Cruz-Santos, M., Ángeles-García, S., Girón-García, P. (2021). Characterization of volcanic ashes deposited in Puebla City. *Tópicos de Investigación en Ciencias de la Tierra y Materiales,**8*(8), 65–76. 10.29057/aactm.v8i8.7667 10.29057/aactm.v8i8.7667

[CR14] Damby, D. E., Murphy, F. A., Horwell, C. J., Raftis, J., & Donaldson, K. (2016). The in vitro respiratory toxicity of cristobalite-bearing volcanic ash. *Environmental Research,**145*, 74–84. 10.1016/j.envres.2015.11.020 26630620 10.1016/j.envres.2015.11.020

[CR15] Dat, N. D., Nguyen, V. T., Vo, T. D. H., Bui, X. T., Bui, M. H., Nguyen, L. S. P., Nguyen, X. C., Tran, A. T. K., Nguyen, T. T. A., Ju, Y. R., & Huynh, T. M. T. (2021). Contamination, source attribution, and potential health risks of heavy metals in street dust of a metropolitan area in Southern Vietnam. *Environmental Science and Pollution Research,**28*, 50405–50419. 10.1007/s11356-021-14246-1 33954920 10.1007/s11356-021-14246-1

[CR16] Edmonds, M., Mason, E., & Hogg, O. (2022). Volcanic outgassing of volatile trace metals. *Annual Review of Earth and Planetary Sciences,**50*, 79–89. 10.1146/annurev-earth-070921-062047 10.1146/annurev-earth-070921-062047

[CR17] Edmonds, M., & Mather, T. A. (2017). Volcanic sulfides and outgassing. *Elements,**13*(2), 105–110. 10.2113/gselements.13.2.105 10.2113/gselements.13.2.105

[CR18] Ermolin, M. S., Fedotov, P. S., Malik, N. A., & Karandashev, V. K. (2018). Nanoparticles of volcanic ash as a carrier for toxic elements on the global scale. *Chemosphere,**200*, 16–22. 10.1016/j.chemosphere.2018.02.089 29471164 10.1016/j.chemosphere.2018.02.089

[CR19] Ermolin, M. S., Ivaneev, A. I., Fedyunina, N. N., & Fedotov, P. S. (2021). Nanospeciation of metals and metalloids in volcanic ash using single particle inductively coupled plasma mass spectrometry. *Chemosphere,**281*, 130950. 10.1016/j.chemosphere.2021.130950 34289616 10.1016/j.chemosphere.2021.130950

[CR20] Expósito, A., Markiv, B., Ruiz-Azcona, L., Santibáñez, M., & Fernández-Olmo, I. (2021). Understanding how methodological aspects affect the release of trace metal(loid)s from urban dust in inhalation bioaccessibility tests. *Chemosphere,**267*, 129181. 10.1016/j.chemosphere.2020.129181 33340883 10.1016/j.chemosphere.2020.129181

[CR21] Ferreira, A. F., Ventura García, P., Camarinho, R., & dos Santos Rodrigues, A. (2015). Volcanogenic pollution and testicular damage in wild mice. *Chemosphere,**132*, 135–141. 10.1016/j.chemosphere.2015.03.017 25828918 10.1016/j.chemosphere.2015.03.017

[CR22] Freire, S., Florczyk, A. J., Pesaresi, M., & Sliuzas, R. (2019). An improved global analysis of population distribution in proximity to active volcanoes, 1975–2015. *International Journal of Geo-Information,**8*(8), 341. 10.3390/ijgi8080341 10.3390/ijgi8080341

[CR23] Gudmundsson, G. (2010). Respiratory health effects of volcanic ash with special reference to Iceland. A Review. *The Clinical Respiratory Journal,**5*(1), 2–9. 10.1111/j.1752-699X.2010.00231.x 21159135 10.1111/j.1752-699X.2010.00231.x

[CR24] Hakanson, L. (1980). An ecological risk index for aquatic pollution control a sedimentological approach. *Water Research,**14*(8), 975–1001. 10.1016/0043-1354(80)90143-8 10.1016/0043-1354(80)90143-8

[CR25] Han, Q., Wang, M., Cao, J., Gui, C., Liu, Y., He, X., He, Y., & Liu, Y. (2020). Health risk assessment and bioaccessibilities of heavy metals for children in soil and dust from urban parks and schools of Jiaozuo, China. *Ecotoxicology and Environmental Safety,**191*, 110157. 10.1016/j.ecoenv.2019.110157 31954218 10.1016/j.ecoenv.2019.110157

[CR26] Henley, R. W., & Berger, B. R. (2013). Nature’s refineries: Metals and metalloids in arc volcanoes. *Earth-Science Reviews,**125*, 146–170. 10.1016/j.earscirev.2013.07.007 10.1016/j.earscirev.2013.07.007

[CR27] Hernández-Pellón, A., Nischkauer, W., Limbeck, A., & Fernández-Olmo, I. (2018). Metal(loid) bioaccessibility and inhalation risk assessment: A comparison between an urban and an industrial area. *Environmental Research,**165*, 140–149. 10.1016/j.envres.2018.04.014 29704775 10.1016/j.envres.2018.04.014

[CR28] Hinkley, T. K., Lamothe, P. J., Wilson, S. A., Finnegan, D. L., & Gerlach, T. M. (1999). Metal emissions form Kilauea, and a suggested revision of the estimated worldwide metal output by quiescent degassing of volcanoes. *Earth and Planetary Science Letters,**170*(3), 315–325. 10.1016/S0012-821X(99)00103-X 10.1016/S0012-821X(99)00103-X

[CR29] Horwell, C. J. (2007). Grain-size analysis of volcanic ash for the rapid assessment of respiratory health hazard. *Journal of Environmental Monitoring,**9*, 1107–1115. 10.1039/B710583P 17909645 10.1039/B710583P

[CR30] Horwell, C. J., & Baxter, P. J. (2006). The respiratory health hazards of volcanic ash: A review for volcanic risk mitigation. *Bulletin of Volcanology,**69*, 1–24. 10.1007/s00445-006-0052-y 10.1007/s00445-006-0052-y

[CR31] Horwell, C. J., Fenoglio, I., Ragnarsdottir, K. V., Sparks, R. S. J., & Fubini, B. (2003). Surface reactivity of volcanic ash from the eruption of Soufrière Hills volcano, Montserrat, West Indies with implications for health hazards. *Environmental Research,**93*(2), 202–215. 10.1016/S0013-9351(03)00044-6 12963405 10.1016/S0013-9351(03)00044-6

[CR32] Ilyinskaya, E., Mason, E., Wieser, P. E., Holland, L., Liu, E. J., Mather, T. A., Edmonds, M., Whitty, R. C., Elias, T., Nadeau, P. A., & Schneider, D. (2021). Rapid metal pollutant deposition from the volcanic plume of Kilauea, Hawaii. *Communications Earth & Environment,**2*, 78. 10.1038/s43247-021-00146-2 10.1038/s43247-021-00146-2

[CR33] Jaishankar, M., Tseten, T., Anbalagan, N., Mathew, B. B., & Beeregowda, K. N. (2014). Toxicity, mechanism and health effects of some heavy metals. *Interdisciplinary Toxicology,**7*(2), 60–72. 10.2478/intox-2014-0009 26109881 10.2478/intox-2014-0009 PMC4427717

[CR34] Kastury, F., Smith, E., Karna, R. R., Scheckel, K. G., & Juhasz, A. L. (2018). Methodological factors influencing inhalation bioaccessibility of metal(loid)s in PM_2.5_ using lung fluid. *Environmental Pollution,**241*, 930–937. 10.1016/j.envpol.2018.05.094 29929159 10.1016/j.envpol.2018.05.094 PMC6517839

[CR36] Kusmartini, I., Syahfitri, W. Y. N., Kurniawati, S., Lestiani, D., & Santoso, M. (2017). Elemental characterization of Mt. Sinabung volcanic ash, Indonesia by neutron activation analysis. *Journal of Physics: Conference Series,**860*, 012005. 10.1088/1742-6596/860/1/012005 10.1088/1742-6596/860/1/012005

[CR37] Lai, R., Oguchi, T., & Zhong, C. (2022). Evaluating spatiotemporal patterns of post-eruption vegetation recovery at Unzen Volcano, Japan, from Landsat Time Series. *Remote Sensing,**14*(21), 5419. 10.3390/rs14215419 10.3390/rs14215419

[CR38] Langmann, B., Folch, A., Hensch, M., & Matthias, V. (2012). Volcanic ash over Europe during the eruption of Eyjafjallajökull on Iceland, April–May 2010. *Atmospheric Environment,**48*, 1–8. 10.1016/j.atmosenv.2011.03.054 10.1016/j.atmosenv.2011.03.054

[CR39] Ling, S., & van Eeden, S. F. (2009). Particulate matter air pollution exposure: Role in the development and exacerbation of chronic obstructive pulmonary disease. *International Journal of Chronic Obstructive Pulmonary Disease,**4*, 233–243. 10.2147/COPD.S5098 19554194 10.2147/COPD.S5098 PMC2699820

[CR40] Lombardo, D., Ciancio, N., Campisi, R., Di Maria, A., Bivona, L., Poletti, V., Mistretta, A., Biggeri, A., & Di Maria, G. (2013). A retrospective study on acute health effects due to volcanic ash exposure during the eruption of Mount Etna (Sicily) in 2002. *Multidisciplinary Respiratory Medicine*. 10.1186/2049-6958-8-51 10.1186/2049-6958-8-51 PMC3750325 23924394

[CR41] Maanan, M., Saddik, M., Maanan, M., Chaibi, M., Assobhei, O., & Zourarah, B. (2015). Environmental and ecological risk assessment of heavy metals in sediments of Nador lagoon, Morocco. *Ecological Indicators,**48*, 616–626. 10.1016/j.ecolind.2014.09.034 10.1016/j.ecolind.2014.09.034

[CR42] Mandon, C. L., Christenson, B. W., Schipper, C. I., Seward, T. M., & Garaebiti, E. (2019). Metal transport in volcanic plumes: A case study at White Island and Yasur volcanoes. *Journal of Volcanology and Geothermal Research,**369*, 155–171. 10.1016/j.jvolgeores.2018.11.024 10.1016/j.jvolgeores.2018.11.024

[CR43] Meza-Figueroa, D., Barboza-Flores, M., Romero, F. M., Acosta-Elias, M., Hernández-Mendiola, E., Maldonado-Escalante, F., Pérez-Segura, E., González-Grijalva, B., Meza-Montenegro, M., García-Rico, L., & Navarro-Espinoza, S. (2020). Metal bioaccessibility, particles size distribution and polydispersity of playground dust in synthetic lysosomal fluids. *Science of the Total Environment,**713*, 136481. 10.1016/j.scitotenv.2019.136481 31954252 10.1016/j.scitotenv.2019.136481

[CR44] Mueller, W., Cowie, H., Horwell, C. J., Hurley, F., & Baxter, P. J. (2020). Health impact assessment of volcanic ash inhalation: A comparison with outdoor air pollution methods. *GeoHealth,**4*(7), e2020GH00256. 10.1029/2020GH000256 10.1029/2020GH000256 PMC7334379 32642627

[CR45] Müller, G. (1969). Index of geoaccumulation in sediments of the Rhine River. *GeoJournal,**2*, 108–118.

[CR46] National Academies of Sciences, Engineering, and Medicine. (2024). *Health risks of indoor exposure to fine particulate matter and practical mitigation solutions*. The National Academies Press. 10.17226/27341 38320080

[CR47] Navarro-Sempere, A., Martínez-Peinado, P., Rodrigues, A. S., Garcia, P. V., Camarinho, R., García, M., & Segovia, Y. (2021). The health hazard of volcanoes: First evidence of neuroinflammation in the hippocampus of mice exposed to active volcanic surrounding. *Mediators of Inflammation*. 10.1155/2021/5891095 10.1155/2021/5891095 PMC8523235 34671225

[CR48] Navarro-Sempere, A., Segovia, Y., Rodrigues, A. S., Garcia, P. V., Camarinho, R., & García, M. (2020). First record on mercury accumulation on mice brain living in active volcanic environments: A cytochemical approach. *Environmental Geochemistry and Health,**43*, 171–183. 10.1007/s10653-020-00690-4 32794111 10.1007/s10653-020-00690-4

[CR49] Paredes-Mariño, J., Scheu, B., Montanaro, C., Arciniega-Ceballos, A., Dingwell, D. B., & Perugini, D. (2019). Volcanic ash generation: Effects of componentry, particle size and conduit geometry on size-reduction processes. *Earth and Planetary Science Letters,**514*, 13–27. 10.1016/j.epsl.2019.02.028 10.1016/j.epsl.2019.02.028

[CR50] Pickarski, N., Kwiecien, O., & Litt, T. (2023). Volcanic impact on terrestrial and aquatic ecosystems in the Eastern Mediterranean. *Communications Earth & Environment,**4*, 167. 10.1038/s43247-023-00827-0 10.1038/s43247-023-00827-0

[CR51] Plumlee, G. S., & Ziegler, T. L. (2007). The medical geochemistry of dusts, soils, and other earth materials. In H. D. Holland & K. K. Turekian (Eds.), *Treatise on geochemistry* (1st ed., pp. 1–61). Elsevier Science. 10.1016/B0-08-043751-6/09050-2

[CR52] Ramos Jiménez, E. (2019). A vulnerability-based risk assessment of the threatened area surrounding Popocatépetl Volcano to support decision-making during a volcanic crisis. *Geofísica Internacional,**58*(1), 7–32. 10.22201/igeof.00167169p.2019.58.1.2064 10.22201/igeof.00167169p.2019.58.1.2064

[CR53] Rodriguez-Espinosa, P. F., Jonathan, M. P., Morales-García, S. S., Campos-Villegas, L. E., Martínez-Tavera, E., Muñoz-Sevilla, N. P., & Alvarado-Corona, M. (2015). Metal enrichment of soils following the April 2012–2013 eruptive activity of the Popocatépetl volcano, Puebla, Mexico. *Environmental Monitoring and Assessment,**187*, 717. 10.1007/s10661-015-4938-z 26514800 10.1007/s10661-015-4938-z

[CR54] Ruggieri, F., Fernández-Turiel, J.-L., Saavedra, J., Gimeno, D., Polanco, E., & Naranjo, J. A. (2011). Environmental geochemistry of recent volcanic ashes from the Southern Andes. *Environmental Chemistry,**8*(3), 236–247. 10.1071/EN10097 10.1071/EN10097

[CR55] Ruggieri, F., Saavedra, J., Fernández-Turiel, J.-L., Gimeno, D., & Garcia-Valles, M. (2010). Environmental geochemistry of ancient volcanic ashes. *Journal of Hazardous Materials,**183*(1–3), 353–365. 10.1016/j.jhazmat.2010.07.032 20675046 10.1016/j.jhazmat.2010.07.032

[CR56] Salamah, S., & Wahyuni, E. T. (2018). The characterization of Merapi volcanic ash as adsorbent for dyes removal from batik wastewater. *IOP Conference Series: Materials Science and Engineering,**403*, 012007. 10.1088/1757-899X/403/1/012007 10.1088/1757-899X/403/1/012007

[CR57] Schaaf, P., Stimac, J., Siebe, C., & Macías, J. L. (2005). Geochemical evidence for mantle origin and crustal processes in volcanic rocks from Popocatépetl and surrounding monogenetic volcanoes, Central Mexico. *Journal of Petrology,**46*(6), 1243–1282. 10.1093/petrology/egi015 10.1093/petrology/egi015

[CR58] Schiavo, B., Meza-Figueroa, D., Pedroza-Montero, M., Vidal-Solano, J., González-Grijalva, B., Navarro-Espinoza, S., Romero, F., Hernández, E., Gutiérrez-Ruiz, M. E., & Ceniceros-Gómez, A. E. (2021). In vitro assessment oral and respiratory bioaccessibility of Mn in school dust: Insight of seasonality in a semiarid environment. *Applied Geochemistry,**134*, 105102. 10.1016/j.apgeochem.2021.105102 10.1016/j.apgeochem.2021.105102

[CR59] Schiavo, B., Meza-Figueroa, D., Vizuete-Jaramillo, E., Robles-Morua, A., Angulo-Molina, A., Reyes-Castro, P. A., Inguaggiato, C., Gonzalez-Grijalva, B., & Pedroza-Montero, M. (2023a). Oxidative potential of metal-polluted urban dust as a potential environmental stressor for chronic diseases. *Environmental Geochemistry and Health,**45*, 3229–3250. 10.1007/s10653-022-01403-9 36197533 10.1007/s10653-022-01403-9

[CR60] Schiavo, B., Morton-Bermea, O., Meza-Figueroa, D., Acosta-Elías, M., González-Grijalva, B., Armienta-Hernández, M. A., Inguaggiato, C., & Valera-Fernández, D. (2023b). Characterization and polydispersity of volcanic Ash nanoparticles in synthetic lung fluid. *Toxics,**11*(7), 624. 10.3390/toxics11070624 37505589 10.3390/toxics11070624 PMC10383943

[CR61] Schiavo, B., Morton-Bermea, O., Salgado-Martinez, E., Arellano, J., & Hernández-Álvarez, E. (2020a). Estimates of mercury flux and temporal variability of Hg/SO_2_ ratio in the plume of Popocatépetl volcano (Mexico). *Journal of South American Earth Sciences,**101*, 102614. 10.1016/j.jsames.2020.102614 10.1016/j.jsames.2020.102614

[CR62] Schiavo, B., Morton-Bermea, O., Salgado-Martinez, E., & Hernández-Álvarez, E. (2020b). Evaluation of possible impact on human health of atmospheric mercury emanations from the Popocatépetl volcano. *Environmental Geochemistry and Health,**42*, 3717–3729. 10.1007/s10653-020-00610-6 32508002 10.1007/s10653-020-00610-6

[CR63] Schiavo, B., Stremme, W., Grutter, M., Campion, R., Guarin, C. A., Rivera, C., & Inguaggiato, S. (2019). Characterization of a UV camera system for SO_2_ measurements from Popocatépetl Volcano. *Journal of Volcanology and Geothermal Research,**370*, 82–94. 10.1016/j.jvolgeores.2018.09.001 10.1016/j.jvolgeores.2018.09.001

[CR64] Stremme, W., Grutter, M., Baylón, J., Taquet, N., Bezanilla, A., Plaza-Medina, E., Schiavo, B., Rivera, C., Blumenstock, T., & Hase, F. (2023). Direct solar FTIR measurements of CO_2_ and HCl in the plume of Popocatépetl Volcano, Mexico. *Frontiers in Earth Science*. 10.3389/feart.2023.1022976 10.3389/feart.2023.1022976

[CR65] Sun, C., Plunkett, G., Liu, J., Zhao, H., Sigl, M., McConnell, J. R., Pilcher, J. R., Vinther, B., Steffensen, J. P., & Hall, V. (2014). Ash from Changbaishan Millennium eruption recorded in Greenland ice: Implications for determining the eruption’s timing and impact. *Geophysical Research Letters,**41*(2), 694–701. 10.1002/2013GL058642 10.1002/2013GL058642

[CR66] Taquet, N., Stremme, W., Grutter, M., Bezanilla, A., Schiavo, B., Rivera, C., Campion, C., Boulesteix, T., Torres, A. N., Perena, R. E., Blumenstock, T., & Hace, F. (2019). Variability in the gas composition of the Popocatepetl volcanic plume. *Frontiers in Earth Science,**7*, 114. 10.3389/feart.2019.00114 10.3389/feart.2019.00114

[CR67] Taylor, H. E., & Lichte, F. E. (1980). Chemical composition of Mount St. Helens Volcanic Ash. *Geophysical Research Letters,**7*(11), 949–952. 10.1029/GL007i011p00949 10.1029/GL007i011p00949

[CR68] Taylor, S. R., & McLennan, S. M. (1985). *The continental crust: Its composition and evolution: An examination of the geochemical record preserved in sedimentary rocks* (Vol. 312). Blackwell science.

[CR69] Thangavel, P., Park, D., & Young-Chul, L. (2022). Recent insights into particulate matter (PM_2.5_)-mediated toxicity in humans: An overview. *International Journal of Environmental Research and Public Health,**19*(12), 7511. 10.3390/ijerph19127511 35742761 10.3390/ijerph19127511 PMC9223652

[CR70] Tomašek, I., Damby, D. E., Stewart, C., Horwell, C. J., Plumlee, G., Ottley, C. J., Delmelle, P., Morman, S., El Yazidi, S., Claeys, P., & Kervyn, M. (2021). Development of a simulated lung fluid leaching method to assess the release of potentially toxic elements form volcanic ash. *Chemosphere,**278*, 130303. 10.1016/j.chemosphere.2021.130303 33819884 10.1016/j.chemosphere.2021.130303

[CR71] Tomasi, C., & Lupi, A. (2016). Primary and secondary sources of atmospheric aerosol. In *Atmospheric aerosols: Life cycles and effects on air quality and climate* (pp. 1–86). 10.1002/9783527336449.ch1

[CR72] Tomii, Y., Shibayama, T., Nishida, Y., Nakamura, R., Okumura, N., Yamaguchi, H., Tanokura, Y., Oshima, Y., Sugawara, N., Fujisawa, K., & Wakita, T. (2020). Estimation of volcanic ashfall deposit and removal works based on ash dispersion simulations. *Natural Hazard,**103*, 3377–3399. 10.1007/s11069-020-04134-1 10.1007/s11069-020-04134-1

[CR101] Trejos, E.M., Silva, L.F.O., Hower, J.C., Flores, E.M.M., González, C.M., Pachón, J.E., Aristizábal, B.H. (2021). Volcanic emissions and Atmospheric Pollution: A study of nanoparticles. *Geoscience Frontiers,**12*(2), 746–755. 10.1016/j.gsf.2020.08.013 10.1016/j.gsf.2020.08.013

[CR73] USEPA. (2001). Risk assessment guidance for superfund: Volume III—Part A, Process for conducting probabilistic risk assessment (EPA 540-R-02-002) (p. 2001). US Environmental Protection Agency. https://www.epa.gov/sites/default/files/2015-09/documents/rags3adt_complete.pdf

[CR74] USEPA. (2010). Integrated Risk Information System (IRIS); United States Environmental Protection Agency. USEPA. United States Environmental Protection Agency. https://cfpub.epa.gov/ncea/risk/recordisplay.cfm?deid=2776

[CR75] Varrica, D., Tamburo, E., Dongarrá, G., & Sposito, F. (2014). Trace elements in scalp hair of children chronically exposed to volcanic activity (Mt. Etna, Italy). *Science of the Total Environment,**470–471*, 117–126. 10.1016/j.scitotenv.2013.09.058 10.1016/j.scitotenv.2013.09.058 24126132

[CR76] Vigneri, R., Malandrino, P., Gianì, F., Russo, M., & Vigneri, P. (2017). Heavy metals in the volcanic environment and thyroid cancer. *Molecular and Cellular Endocrinology,**457*, 73–80. 10.1016/j.mce.2016.10.027 27794445 10.1016/j.mce.2016.10.027

[CR77] Werner, C., Janik, C. J., Goff, F., Counce, D., Johnson, L., Siebe, C., Delgado, H., Williams, S. N., & Fischer, T. P. (1997). Geochemistry of summit fumarole vapors and flanking therma/mineral waters at Popocatépetl Volcano, Mexico. Los Alamos National Laboratory report, LA-13289-MS, 33 pp. 10.2172/495722

[CR78] Witter, J. B., Kress, V. C., & Newhall, C. G. (2005). Volcán Popocatépetl, Mexico. Petrology, Magma mixing, and immediate sources of volatiles for the 1994: Present eruption. *Journal of Petrology,**46*(11), 2337–2366. 10.1093/petrology/egi058 10.1093/petrology/egi058

[CR79] Woitischek, J., Mingotti, N., Edmonds, M., & Woods, A. W. (2021). On the use of plume models to estimate the flux in volcanic gas plumes. *Nature Communications,**12*, 2719. 10.1038/s41467-021-22159-3 10.1038/s41467-021-22159-3 PMC8113474 33976131

[CR80] Yongming, H., Peixuan, D., Junji, C., & Posmentier, E. S. (2008). Multivariate analysis of heavy metal contamination in urban dusts of Xi’an, Central China. *Science of the Total Environment,**335*(1–3), 176–186. 10.1016/j.scitotenv.2005.02.026 10.1016/j.scitotenv.2005.02.026 15885748

[CR81] Zhang, J., Chen, Z., Shan, D., Wu, Y., Zhao, Y., Li, C., Shu, Y., Linghu, X., & Wang, B. (2024). Adverse effects of exposure to fine particles and ultrafine particles in the environment on different organs of organisms. *Journal of Environmental Sciences,**135*, 449–473. 10.1016/j.jes.2022.08.013 10.1016/j.jes.2022.08.013 37778818

