# Supplementary material for: Metal(loid) bioaccessibility and risk assessment of ashfall deposit from Popocatépetl volcano, Mexico
Source: Environ Geochem Health. 2024 Jul 30;46(9):354. doi: 10.1007/s10653-024-02135-8 (PMC11289158; doi:10.1007/s10653-024-02135-8)
Supplement: Supplementary file 1 — Supplementary file1 (DOCX 1273 KB) [file 10653_2024_2135_MOESM1_ESM.docx]

**Metal(loid) bioaccessibility and risk assessment of ashfall**

**deposits from Popocatépetl volcano, Mexico**

Benedetto Schiavo^1*^, Diana Meza-Figueroa^2^, Ofelia Morton-Bermea^1^, Araceli Angulo-Molina^3^, Belem González-Grijalva^2^, Maria Aurora Armienta-Hernández^1^, Claudio Inguaggiato^4^, Francisco Berrellez-Reyes^2^, Daisy Valera-Fernández^5^

*^1^Instituto de Geofísica, Universidad Nacional Autónoma de México, 04510, Mexico City, Mexico*

*^2^Departamento de Geología, Universidad de Sonora, Hermosillo 83000, Mexico*

*^3^Departamento de Ciencias Químico Biológicas, Universidad de Sonora, Hermosillo 83000, Mexico*

*^4^Departamento de Geología, Centro de Investigación Científica y de Educación Superior de Ensenada, Baja California (CICESE), Ensenada, Mexico*

*^5^Instituto de Geología, Universidad Nacional Autónoma de México, 04510, Mexico City, Mexico*

*Corresponding author:

Benedetto Schiavo, [benedetto@igeofisica.unam.mx](mailto:benedetto@igeofisica.unam.mx), ORCID: 0000-0002-0882-0404

**Figure S1:** Location of sampling points at Popocatépetl volcano.


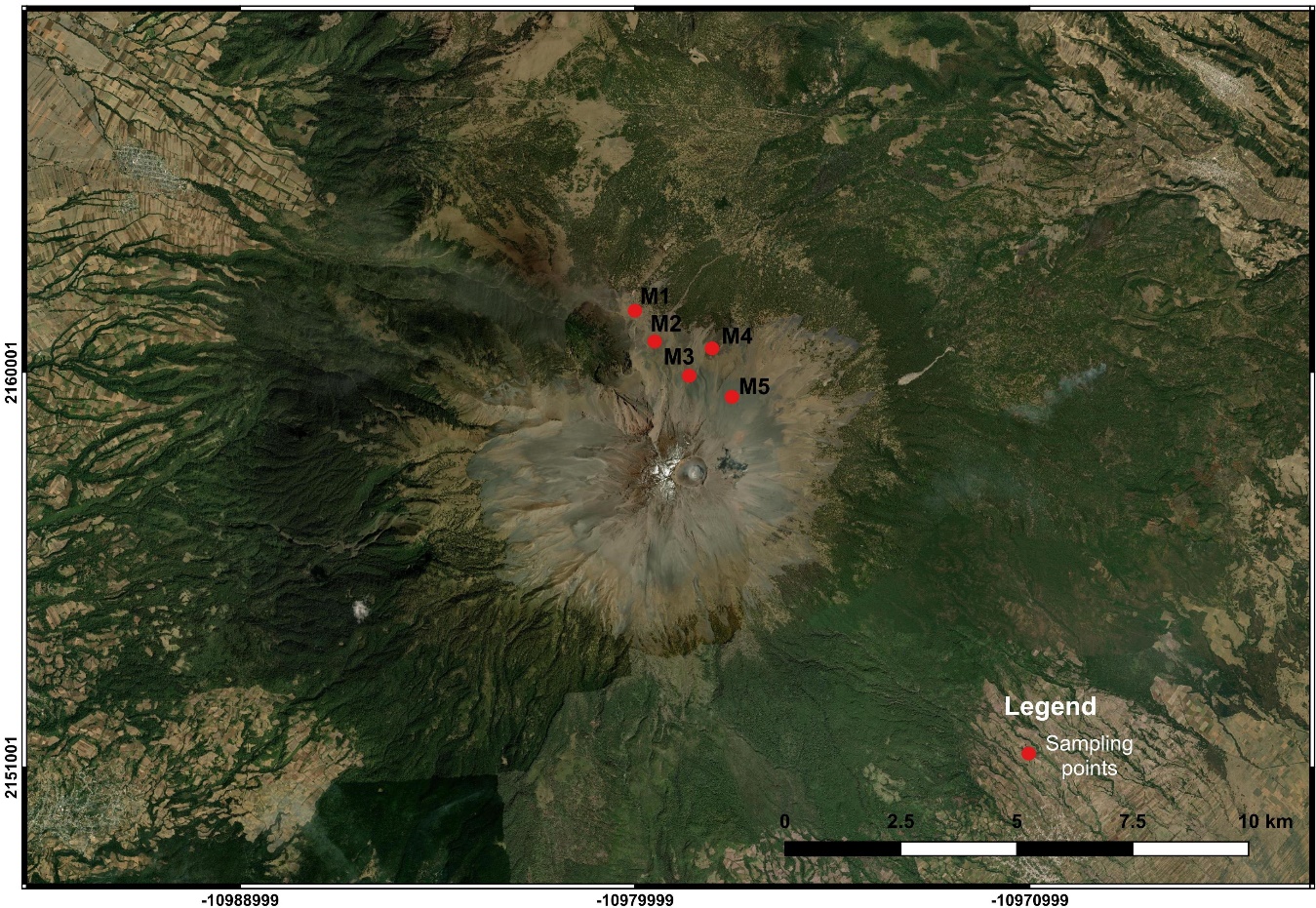


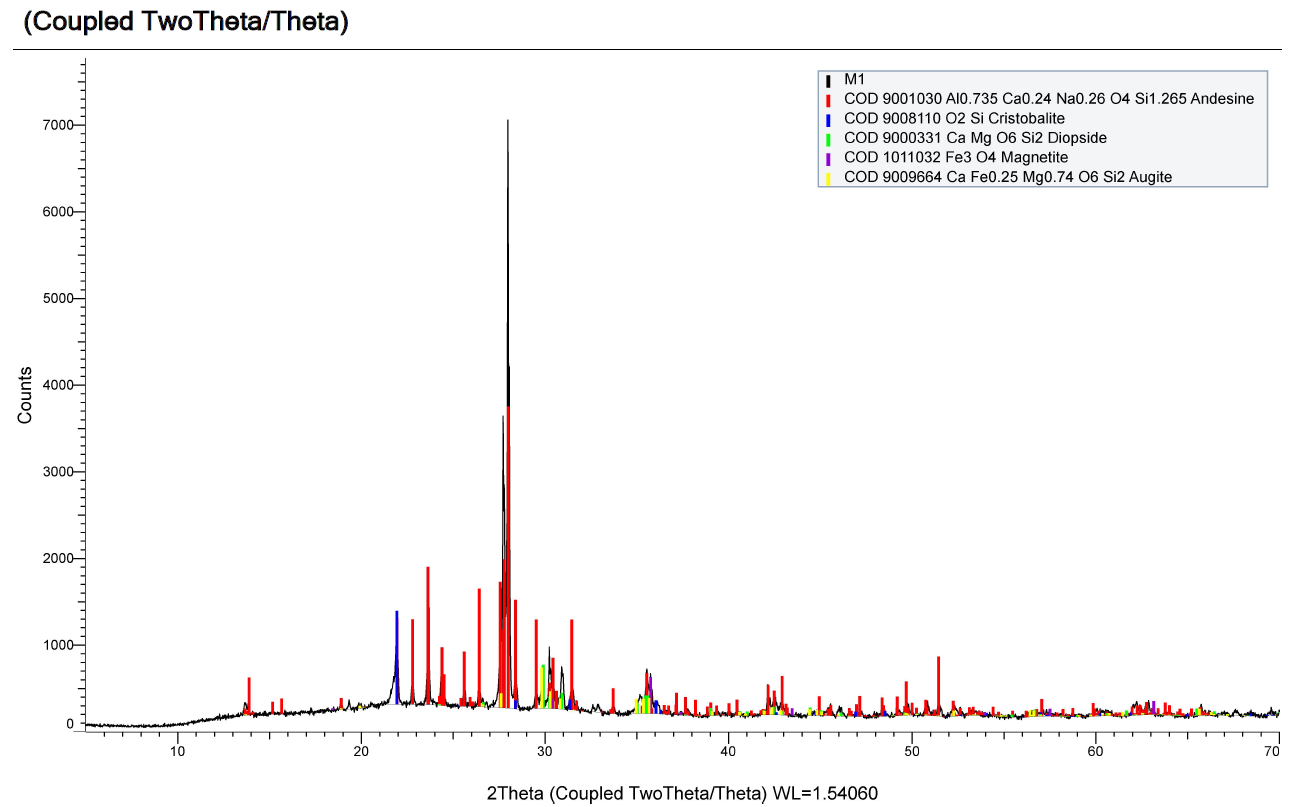
**Figure S2:** Mineralogical phases recognized in ashfall samples from XRD analysis. A) M1, B) M2, C) M3, D) M4, and E) M5.

**A**

**B**

**CB**

**D**

**ED**

**Table S1:** Quality control of results obtained with ICP-MS using SRM-2709A.

|  | **SRM-2709A** | **ICP-MS** |
| --- | --- | --- |
| **Metal(loid)**  **(mg kg^-1^)** |  |  |
| As | 10.5 | 8.9 |
| Cd | 0.37 | 0.38 |
| Cr | 130 | 160.6 |
| Cu | 33.9 | 35.6 |
| Mn | 529 | 657.2 |
| Ni | 85 | 97.8 |
| Pb | 17.3 | 21.5 |
| Zn | 103 | 96.9 |

**Table S2:** Classification and description of geo-accumulation index (I_geo_), enrichment factor (EF), and potential ecological risk (E_r_).

| **Index** | **Classification** | **Description** |
| --- | --- | --- |
| Geo-accumulation | I_geo_$\leq$0 | Uncontaminated |
|  | 0$<$ I_geo_$\leq$1 | Uncontaminated to moderate contaminated |
|  | $1<$ I_geo_$\leq$2 | Moderate contaminated |
|  | $2<$ I_geo_$\leq$3 | Moderate to strong contaminated |
|  | $3<$ I_geo_$\leq$4 | Strong contaminated |
|  | $4<$ I_geo_$\leq$5 | Strong to extreme contaminated |
|  | I_geo_$>$5 | Extreme contaminated |
| Enrichment factor | EF$<$1 | No enrichment |
|  | 1$\leq$EF$\leq$3 | Minor enrichment |
|  | 3$\leq$EF$\leq$5 | Moderate enrichment |
|  | 5$\leq$EF$\leq$10 | Moderate to severe enrichment |
|  | 10$\leq$EF$\leq$25 | Severe enrichment |
|  | 25$\leq$EF$\leq$50 | Very severe enrichment |
|  | EF$\geq$50 | Extreme enrichment |
| Potential ecological risk | $RI<$40 | Low potential ecological risk |
|  | 40$<RI<$80 | Moderate potential ecological risk |
|  | 80$<RI<$160 | Considerable potential ecological risk |
|  | 160$<RI<$320 | High potential ecological risk |
|  | $RI>$320 | Very high potential ecological risk |

**Table S3:** Parameters and values for human health risk calculation (USEPA, 2002; Cakmak et al., 2020).

|  |  |  | Value | |
| --- | --- | --- | --- | --- |
| Factor | Unit | Definition | Children | Adults |
| ADD | mg kg^-1^ day^-1^ | Average daily dose through  ingestion, inhalation, and dermal contact | - | - |
| C | mg kg^-1^ | Concentration of metal(oid) | - | - |
| IngR | mg day^-1^ | Ingestion rate | 200 | 100 |
| InhR | m^3^ day^-1^ | Inhalation rate | 7.6 | 20 |
| EF | days year^-1^ | Exposure frequency | 350 | 350 |
| ED | years | Exposure duration | 6 | 24 |
| BW | kg | Body weight | 10 | 55 |
| AT_nc_ | days | Average time (for non-carcinogens) | 365×ED | 365×ED |
| AT_c_ | days | Average time (for carcinogens) | 365×70 | 365×70 |
| CF | kg mg^-1^ | Conversion factor | 1×10^-6^ | 1×10^-6^ |
| SA | cm^2^ | Surface skin area available  for exposure | 2800 | 5700 |
| AF | mg cm^-2^ day^-1^ | Skin adherence factor | 0.2 | 0.07 |
| ABF | - | Dermal adsorption factor | 0.001 | 0.001 |
| PEF | m^3^ kg^-1^ | Particle emission factor | 1.36×10^9^ | 1.36×10^9^ |

**Table S4:** Reference dose (RfD, mg kg^-1^ day^-1^) values for non-carcinogenic risk and slope factor (SF, kg mg^-1^ day^-1^) values for carcinogenic risk (USEPA, 2002; USEPA, 2010; Cakmak et al., 2020; Dat et al., 2021; Ferreira-Baptista and De Miguel, 2005; Khan et al., 2023).

| **Metal(loid)** |  | **RfD** |  |  |  | **SF** |  |
| --- | --- | --- | --- | --- | --- | --- | --- |
|  | Ingestion | Inhalation | Dermal contact |  | Ingestion | Inhalation | Dermal contact |
| As | 3.0E-04 | 5.0E-04 | 1.2E-04 |  | 1.5E+00 | 1.5E+01 | 3.7E+00 |
| Cd | 1.0E-03 | 1.0E-05 | 2.5E-05 |  | - | 6.3E+00 | - |
| Cr | 3.0E-03 | 2.7E-05 | 1.9E-02 |  | 5.0E-01 | 4.1E+01 | - |
| Cu | 4.0E-02 | 1.2E-02 | 4.0E-02 |  | - | - | - |
| Mn | 2.4E-02 | 5.0E-05 | 9.6E-04 |  | - | - | - |
| Ni | 2.0E-02 | 5.4E-03 | 2.0E-02 |  | - | 8.4E-01 | - |
| Pb | 1.4E-03 | 3.52E-03 | 1.4E-03 |  | 8.5E-03 | 4.2E-02 | - |
| Zn | 3.0E-01 | 3.5E+01 | 3.0E-01 |  | - | - | - |

**Table S5:** Chemical composition of GS and ALF solutions used for in vitro lung bioaccessibility test (Meza-Figueroa et al, 2020).

| Reagent required for 1 L of solution (g) | Formula | GS  (pH~7) | ALF  (pH~4.5) |
| --- | --- | --- | --- |
| Magnesium chloride  hexahydrate | MgCl_2_·6H_2_O | 0.10 | 0.05 |
| Sodium chloride | NaCl | 6.02 | 3.21 |
| Potassium chloride | KCl | 0.30 | - |
| Disodium hydrogen phosphate | Na_2_HPO_4_ | 0.13 | 0.07 |
| Sodium sulphate | Na_2_SO_4_ | 0.06 | 0.04 |
| Calcium chloride dihydrate | CaCl_2_·2H_2_O | 0.37 | 0.13 |
| Sodium acetate | C_2_H_3_O_2_Na | 0.57 | - |
| Sodium hydrogen carbonate | NaHCO_3_ | 2.6 | - |
| Sodium citrate dihydrate | C_6_H_5_Na_3_O_7_·2H_2_O | 0.10 | 0.08 |
| Sodium hydroxide | NaOH | - | 6 |
| Citric acid | C_6_H_8_O_7_ | - | 20.8 |
| Glycine | H_2_NCH_2_COOH | - | 0.06 |
| Sodium tartrate dihydrate | C_4_H_4_O_6_Na_2_·2H_2_O | - | 0.09 |
| Sodium lactate | C_2_H_5_NaO_3_ | - | 0.09 |
| Sodium pyruvate | C_3_H_3_O_3_Na | - | 0.09 |

**Table S6:** Average daily dose (ADD, mg kg^-1^ day^-1^) values of metal(loid) for non-carcinogenic risk in children and adults.

| **Metal(loid)** | **ADD_ing_** |  |  | **ADD_inh_** |  |  | **ADD_der_** |  |  | **ADD** |  |
| --- | --- | --- | --- | --- | --- | --- | --- | --- | --- | --- | --- |
|  | Children | Adults |  | Children | Adults |  | Children | Adults |  | Children | Adults |
| As | 2.5E-05 | 2.7E-06 |  | 7.1E-10 | 4.0E-10 |  | 7.1E-08 | 1.2E-08 |  | 2.5E-05 | 2.7E-06 |
| Cd | 2.1E-06 | 2.2E-07 |  | 5.8E-11 | 3.3E-11 |  | 5.8E-09 | 8.9E-10 |  | 2.1E-06 | 2.2E-07 |
| Cr | 1.7E-03 | 1.8E-04 |  | 4.8E-08 | 2.7E-08 |  | 4.8E-06 | 7.3E-07 |  | 1.7E-03 | 1.8E-04 |
| Cu | 1.1E-04 | 1.2E-05 |  | 3.1E-09 | 1.7E-09 |  | 3.1E-07 | 4.7E-08 |  | 1.1E-04 | 1.2E-05 |
| Mn | 8.9E-03 | 9.6E-04 |  | 2.5E-07 | 1.4E-07 |  | 2.5E-05 | 3.8E-06 |  | 8.9E-03 | 9.6E-04 |
| Ni | 7.1E-04 | 7.6E-05 |  | 2.0E-08 | 1.1E-08 |  | 2.0E-06 | 3.0E-07 |  | 7.1E-04 | 7.6E-05 |
| Pb | 1.1E-04 | 1.2E-05 |  | 3.1E-09 | 1.8E-09 |  | 3.1E-07 | 4.8E-08 |  | 1.2E-04 | 1.3E-05 |
| Zn | 1.3E-03 | 1.4E-04 |  | 3.7E-08 | 2.1E-08 |  | 3.7E-06 | 5.7E-07 |  | 1.3E-03 | 1.4E-04 |

**References**

Čakmak, D., Perović, V., Kresović, M. et al. (2020). Sources and a Health Risk Assessment of Potentially Toxic Elements in Dust at Children’s Playgrounds with Artificial Surfaces: A Case Study in Belgrade. Arch Environ Contam Toxicol 78, 190–205. <https://doi.org/10.1007/s00244-019-00702-0>

Dat, N.D., Nguyen, VT., Vo, TDH. et al. (2021). Contamination, source attribution, and potential health risks of heavy metals in street dust of a metropolitan area in Southern Vietnam. Environ Sci Pollut Res 28, 50405–50419.

[https://doi.org/10.1007/s11356- 021-14246-1](https://doi.org/10.1007/s11356-%09021-14246-1)

Ferreira-Baptista, L., and De Miguel, E. (2005). Geochemistry and risk Assessment od street dust in Luanda, Angola: A tropical urban environment. Atmospheric Environment, 39, 4501-4512. <https://doi.org/10.1016/j.atmosenv.2005.03.026>

Khan, M., Setu, S., Sultana, N. et al. (2023). Street dust in the largest urban agglomeration: pollution characteristics, source apportionment and health risk assessment of potentially toxic trace elements. Stoch Environ Res Risk Assess 37, 3305–3324. <https://doi.org/10.1007/s00477-023-02432-1>

Meza-Figueroa, D., Barboza-Flores, M., Romero, F.M., et al. (2020). Metal bioaccessibility, particles size distribution and polydispersity of playground dust in synthetic lysosomal fluids. Science of The Total Environment, 713, 136481.

<https://doi.org/10.1016/j.scitotenv.2019.136481>

USEPA (2002) Supplemental Guidance for Developing Soil Screening Levels for Superfund Sites. The U.S. Environmental Protection Agency, Office of Emergency and Remedial Response, Washington, DC.

USEPA (2010) Integrated Risk Information System (IRIS); United States Environmental Protection Agency. USEPA (United States Environmental Protection Agency)., Washington.
